# Differentiation-associated ISG expression of NK cells in chronic viral infection

**DOI:** 10.1016/j.isci.2025.113216

**Published:** 2025-07-26

**Authors:** Franziska Keller, Robert Lorenz Chua, Timo Trefzer, Katharina Jechow, Liane Bauersfeld, Fabian Beier, Sagar, Özlem Sogukpinar, Giuseppe Rusignuolo, Marta Rizzi, Roland Eils, Andreas Pichlmair, Marco Binder, Bertram Bengsch, Christoph Neumann-Haefelin, Volker Lohmann, Tobias Boettler, Christian Conrad, Robert Thimme, Maike Hofmann

**Affiliations:** 1 Department of Medicine II (Gastroenterology, Hepatology, Endocrinology and Infectious Diseases), Medical Center- University of Freiburg, 79106 Freiburg, Baden-Württemberg, Germany; 2 Faculty of Biology, University of Freiburg, 79104 Freiburg, Baden-Württemberg, Germany; 3 Center for Digital Health, Berlin Institute of Health (BIH) and Charité - Universitätsmedizin Berlin, corporate member of Freie Universität Berlin, Humboldt-Universität zu Berlin, 10178 Berlin, Germany; 4 Department of Rheumatology and Clinical Immunology, Medical Center - University of Freiburg, 79106 Freiburg, Baden-Württemberg, Germany; 5 Technical University of Munich, School of Medicine, Institute of Virology, 81675 Munich, Bavaria, Germany; 6 German Center for Infection Research (DZIF), Munich Partner Site, 81675 Munich, Bavaria, Germany; 7 Research Group “Dynamics of Early Viral Infection and the Innate Antiviral Response”, Division Virus-Associated Carcinogenesis (D430), German Cancer Research Center, 69120 Heidelberg, Baden-Württemberg, Germany; 8 Department of Infectious Diseases, Molecular Virology, Section Virus-Host-Interactions, University of Heidelberg, Heidelberg, Germany; 9 German Centre for Infection Research (DZIF), Partner Site Heidelberg, 69120 Heidelberg, Baden-Württemberg, Germany

**Keywords:** Immunology, Transcriptomics

## Abstract

Natural killer (NK) cell responses are modulated by type-I interferons (IFNs) in viral infection. Chronic hepatitis C virus (HCV) infection, marked by robust IFN signatures, shows NK cells with reduced cytokine release but heightened cytotoxicity. Comparable alterations occur in chronic hepatitis B virus (HBV) infection even without a pronounced IFN milieu, implying additional regulatory layers. We analyzed NK cells from healthy donors and patients with chronic HBV or HCV and found conserved expression patterns of interferon-stimulated genes (ISGs) such as *IFITM3*, *IRF1*, *IFIT2,* and *ISG20* that correlated with NK cell differentiation state. These genes are governed by fate-determining transcription factors, including ETS1, FLI1, and Eomes, and appear to be constitutively expressed rather than driven by persistent IFN exposure. Network analysis suggested that NK cell ISGs participate not only in antiviral defense but also in processes such as transport and metabolism, underscoring their role in shaping NK responses during health and chronic viral infection.

## Highlights

• NK cells intrinsically express some ISGs beyond classical IFN-dependent induction
• Conserved ISG patterns in NK cells from healthy and patients with chronic HBV/HCV
• Intrinsically expressed ISGs link to NK cell differentiation in health and infection
• ISGs link to biological processes in NK cells beyond an antiviral state

## Graphical abstract

**Figure:**
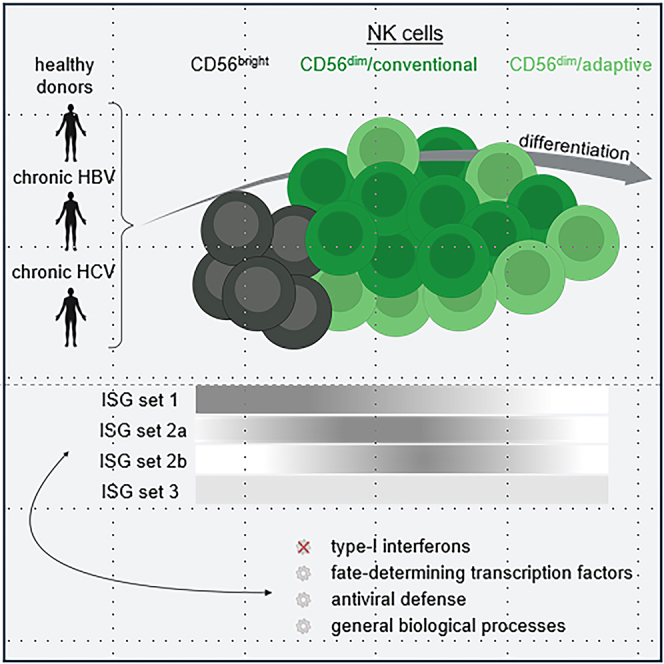

## Introduction

Natural killer (NK) cells are important innate effector cells in controlling viral infections. NK cells are composed of different subsets, in particular CD56^bright^, conventional CD56^dim^, and adaptive CD56^dim^ NK cells, with distinct characteristics of responsiveness and functionality. They quickly act in concert with other innate immune cells to mediate anti-viral immunity.^1^ Besides receptor-based regulation, NK cells are also activated by various cytokines (e.g., Interleukin (IL)-2, IL-12, IL-15, and IL-18) and interferons (IFNs), which are produced by virus-infected cells or antigen-presenting cells.^2^ More specifically, interferons not only activate NK cells but also act as modulators of NK cell functionality. A direct modulating effect of type-I IFNs (IFNα and IFNβ) on NK cells is mediated by binding to the type-I IFN receptor (IFNAR) and activation of the JAK-STAT signaling cascade. This results in the induction of a broad network of interferon-stimulated genes (ISGs), enabling versatile modulating effects.^3^^,^^4^ Strong and persisting IFN signaling during viral infections, such as with hepatitis C virus (HCV), robustly induces cytotoxicity while reducing cytokine production in NK cells by the elevated expression of the ISG STAT1.^1^^,^^5^^,^^6^ In the context of chronic HCV infection, it has also been shown that this functional NK cell phenotype with high cytotoxicity and diminished cytokine production is normalized after anti-viral therapy with direct-acting antivirals (DAA) due to the rapid elimination of HCV, and that this is associated with the abrogation of type-I/III IFN signaling.^7^^,^^8^ This observation highlights an important role of type-I/III IFN in at least partially modulating NK cell functionality in a reversible manner in chronic viral hepatitis. However, the distinct roles of ISGs in NK cell modulation in chronic viral hepatitis and also in healthy donors have not been investigated in detail.

ISGs are primarily described as IFN-dependent genes with a function as positive^9^^,^^10^^,^^11^ or negative^12^^,^^13^ regulators in the anti-viral immune response. They have the potential to directly induce an anti-viral state in cells. For example, ISGs control viral infections by directly targeting pathways and functions required during viral life cycles, or by fostering cellular homeostasis over viral replication. Some ISGs are also involved in general biological processes beyond anti-viral immunity, such as cellular transport, gene expression, and metabolic processes.^14^ In addition to the IFN-dependent regulation of ISGs, recent studies have highlighted that a set of ISGs (including IFITM family members) is intrinsically expressed in human embryonic stem cells (hESCs), independently of IFNs. Indeed, these ISGs are highly enriched in hESCs, and decrease during cell differentiation, setting those cells in a generally protected state.^15^^,^^16^ It is, however, not clear yet whether this intrinsic ISG expression exhibits a role beyond anti-viral immunity, which factors are involved in the intrinsic regulation of ISGs, and whether IFN-independent intrinsic regulation is evident beyond hESCs, for example, in immune cells that mediate anti-viral effector functions such as NK cells.

In this study, we have therefore investigated the expression, regulation, and role of ISGs as potential modulators of NK cell immunity. For this, we performed single cell RNA sequencing (scRNAseq) and comparative protein expression analysis of circulating NK cells in healthy donors (HD) and patients with chronic hepatitis B virus (HBV) and HCV infection. We used HBV for comparing a viral infection that does not induce a significant early IFN response (so-called “stealth” virus) to HCV, a potent interferon inducer.^17^ We uncovered that NK cells express ISGs irrespective of an established ongoing chronic infection with HBV and HCV, beyond IFN-dependent induction and associated with their differentiation state and general biological processes. This observation highlights diverse roles of ISGs in addition to the establishment of an anti-viral cellular state within the immune system and links ISG expression to different NK cell subsets that shape the NK cell response in healthy and during chronic viral infection, and thus also irrespective of an ongoing infection.

## Results

### Distinct interferon-stimulated gene expression in natural killer cell clusters from healthy donors

**Figure 1:**
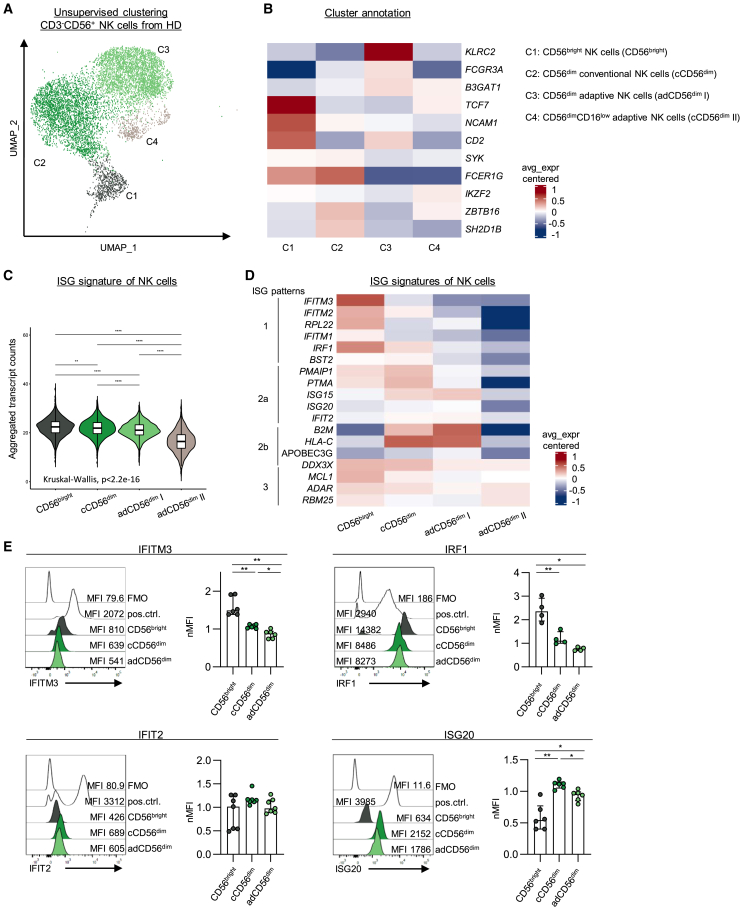
Distinct ISG expression in NK cell clusters from healthy donors ScRNAseq analysis of isolated circulating CD3^−^CD56^+^ NK cells from HCMV^+^/HD (HD1: 14752 cells, HD2: 14595 cells). (A) Unsupervised UMAP cluster analysis showed four distinct NK cell clusters C1-C4 (dark gray: C1, green: C2, light green: C3 and beige: C4). Each dot represents one CD3^−^CD56^+^ NK cell. (B) Heatmap with centered averaged expression of NK cell lineage markers indicates four NK cell clusters as C1 = CD56^bright^ NK cells (CD56^bright^); C2 = CD56^dim^ conventional NK cells (cCD56^dim^); and two clusters with an adaptive phenotype, C3 and C4 = CD56^dim^CD16^low^ adaptive NK cells (adCD56^dim^ I and adCD56^dim^ II). (C and D) ISG expression in CD56^bright^, cCD56^dim^, adCD56^dim^ I and adCD56dim II NK cells. (C) Violin plot with aggregated transcript counts and (D) heatmap with centered averaged expression. (E) Flow cytometric analysis of IFITM3, IRF1, IFIT2 and ISG20 protein expression in NK cells of HCMV^+^/HDs (n = 4–7). Representative flow cytometry histograms and bar graphs depicting the normalized MFI of IFITM3, IRF1, and IFIT2 and ISG20 expression in CD56^bright^, cCD56^dim^, and adCD56^dim^ NK cells. Protein marker expression was normalized to CD3^−^CD56^+^ NK cells. Bar charts indicate the median value with IQR. Statistical analyses were performed (C) via Kruskal-Wallis test, *p* < 2.2^e−16^, and (E) via one-way repeated measures ANOVA (IFITM3, IRF1, ISG20) and Friedmann test (IFIT2), ∗: *p* < 0.05, ∗∗: *p* < 0.01, ∗∗∗∗: *p* < 0.0001. ScRNAseq, single cell RNA sequencing; HCMV^+^, human cytomegalovirus seropositive; HD, healthy donor; UMAP, uniform manifold approximation and projection; ISG, interferon-stimulated gene; MFI, median fluorescence intensity; IQR, interquartile range. See also Figures S1 and S2.

To obtain a comprehensive picture of ISGs expressed by NK cells, we first performed scRNAseq. For this, a total of 24,834 circulating CD3^−^CD56^+^ NK cells were purified from peripheral blood mononuclear cells (PBMCs) of human cytomegalovirus seropositive healthy donors (HCMV^+^/HD) and prepared for scRNAseq (Figures S1A and S1B). Unsupervised clustering revealed four different NK cell (*NCAM*^*+*^*CD3E*^*-*^*CD3G*^*-*^*CD3D*^*-*^*CD19*^*−*^) clusters (Figures 1A, 1B, and S1C). Cluster 1 highly expressed *NCAM1* (encoding for CD56), *CD2*, *TCF7* (encoding for TCF1), while lacking *FCGR3A* (encoding for CD16) expression and thus exhibited a molecular profile characteristic of CD56^bright^ NK cells. Cluster 2 corresponded to conventional CD56^dim^ NK cells (cCD56^dim^) characterized by the reduced expression of *NCAM1* and increased expression of *FCGR3A*. Finally, clusters 3 and 4, both showed features of adaptive NK cells (adCD56^dim^ I and II), including the limited expression of *FCER1G* (encoding for FcεRIγ)*, SYK*, and *SH2D1B*; however, they differed in *FCGR3A*, *IKZF2* (encoding for Helios), and *ZBTB16* (encoding for PLZF) expressions.

ISGs were broadly expressed in the identified circulating NK cell clusters (Figure 1C). However, ISG expression was clearly distinct between NK cell clusters allowing the identification of four different ISG patterns (Figure 1D) assigned to CD56^bright^, cCD56^dim^, adCD56^dim^ I and adCD56^dim^ II NK cells, respectively. In particular, CD56^bright^ NK cells expressed higher levels of *IFITM1/2/3, RPL22, IRF1*, and *BST2* reminiscent of the ISG signature intrinsically expressed by pluripotent stem cells.^16^ Indeed, CD56^bright^ NK cells also showed an enrichment of this previously reported stem-like ISG signature (Figure S1D). cCD56^dim^ NK cells were characterized by ISG pattern 2a (comprising higher levels of e.g., *ISG15, ISG20, and IFIT2*) and 2b while adCD56^dim^ I NK cells mainly expressed ISG pattern 2b including *B2M and HLA-C*. ISG pattern 3 (containing ISGs such as *DDX3X* and *ADAR*) was expressed by all four NK cell clusters, also to a weak extent by adCD56^dim^ II NK cells. Next, to validate the ISG patterning on the protein level, we performed *ex vivo* analysis of NK cells from HCMV^+^/HD by flow cytometry. We used co-expression analysis of CD56, CD16, and FcεRIγ to identify CD56^bright^, cCD56^dim^, and adCD56^dim^ NK cells (Figures S2A and S2B). Of note, we did not observe the matching expression of PLZF, Helios, or NKG2C on protein and RNA levels that allowed further separation of adCD56^dim^ NK cells into adCD56^dim^ I and adCD56^dim^ II according to transcriptome-based cluster analysis. Therefore, ISG protein expression analysis was performed on the entire adCD56^dim^ NK cell population (CD56^dim^, CD16^+^, FcεRIγ^−^). Subsequently, IFITM3 and IRF1 served as surrogate markers for ISG pattern 1; and IFIT2 and ISG20 as surrogates for ISG pattern 2. Similar to the transcriptomic data, IFITM3 and IRF1 were highly expressed in CD56^bright^ NK cells with a gradually decreased expression in cCD56^dim^ NK cells, followed by adCD56^dim^ NK cells (Figure 1E; corresponding gene expression Figure 1D). In contrast, IFIT2 and ISG20 showed slightly higher expression levels in cCD56^dim^ and adCD56^dim^ NK cells compared to CD56^bright^ NK cells (Figure 1E, as a trend or statistically significant, respectively). Hence, differential ISG expression was detectable in different NK cell clusters on a transcriptomic and protein level, with the enriched expression of an ISG signature in CD56^bright^ NK cells that was previously described in the context of pluripotent stem cells.

### Conserved ISG patterns in NK cells during chronic hepatitis B and hepatitis C infection

**Figure 2:**
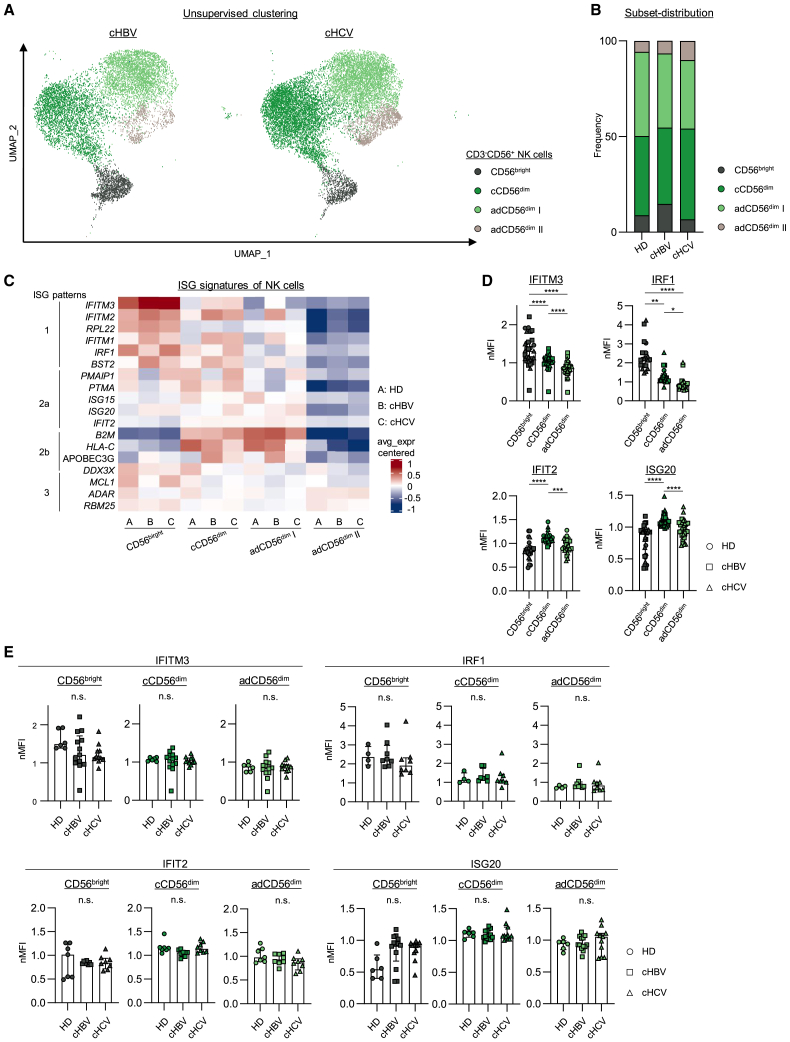
Conserved ISG patterns in NK cells during chronic viral infection Comparative scRNAseq analysis of isolated circulating CD3^−^CD56^+^ NK cells from patients suffering from HCMV^+^/cHBV or HCMV^+^/cHCV infection (HBV1: 9760 cells, HBV2: 9921 cells, HBV3: 10030 cells, HCV1: 9957 cells, HCV2: 9923 cells, HCV3: 9831 cells). (A) Unsupervised UMAP cluster analysis to identify NK cell clusters (dark gray: CD56^bright^, green: cCD56^dim^, light green: adCD56^dim^ I and beige: adCD56^dim^ II) in HCMV^+^/cHBV- and HCMV^+^/cHCV-infected patients. Each dot represents one CD3^−^CD56^+^ NK cell. (B) Bar graph with frequencies of NK cell cluster distribution on RNA level in HCMV^+^/HDs, HCMV^+^/cHBV-, and HCMV^+^/cHCV-infected patients is depicted. (C) Centered average expression of ISGs in NK cell clusters from HCMV^+^/HD, HCMV^+^/cHBV-, and HCMV^+^/cHCV-infected patients is shown. (D) Normalized MFI of IFITM3, IRF1, IFIT2 and ISG20 on protein level in NK cell clusters obtained from HCMV^+^/cHBV- (n = 8–13)or HCMV^+^/cHCV-infected patient (n = 8–11)and from HCMV^+^/HDs (n = 4–7). (E) Normalized MFI of IFITM3, IRF1, IFIT2, and ISG20 on protein level in NK cells separated by subsets. Bar charts indicate the median value with IQR. MFI of ISG expression was normalized to CD3^−^CD56^+^ NK cells. Statistical significance was assessed by: (D) ordinary one-way repeated measures ANOVA (IFITM3, ISG20) and Friedmann test (IRF1, IFIT2); and (E) RM one-way repeated measures ANOVA (IFITM3, ISG20), Kruskal-Wallis test (IRF1/CD56^bright^/cCD56^dim^, IFIT2/CD56^bright^) and Friedmann test (IRF1/adCD56^dim^, IFIT2/cCD56^dim^/adCD56^dim^. *p* > 0.05, ∗: *p* < 0.05, ∗∗: *p* < 0.01, ∗∗∗: *p* < 0.001, ∗∗∗∗: *p* < 0.0001. cHBV, chronic hepatitis B virus infection; cHCV, chronic hepatitis C virus infection. n.s., not significant. See also Figure S3.

**Figure 3:**
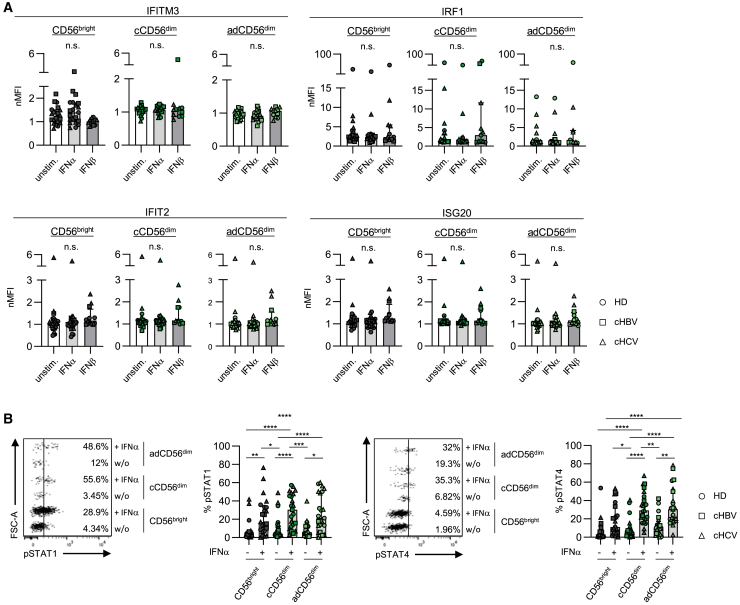
Impact of type-I IFNs on ISG expression in NK cells Impact of IFNα and IFNβ on NK cell clusters (CD56^bright^, cCD56^dim^ and adCD56^dim^ NK cells) from HCMV^+^/HD, HCMV^+^/cHBV- and HCMV^+^/cHCV-infected patients. (A) Bar graphs depicting the normalized MFI of ISG expression upon or without (unstim.) IFN stimulation overnight in NK cell clusters from HCMV^+^/HD (n = 3–6), HCMV^+^/cHBV- (n = 3–9), and HCMV^+^/cHCV-infected patients (n = 5–8). (B) Representative flow cytometry dot plots show the induction of pSTAT1 and pSTAT4 after IFNα2 stimulation for 3 h. Bar charts indicate frequencies of pSTAT1 and pSTAT4 expression with or without IFNα2 stimulation in NK cell clusters from HCMV^+^/HDs (*n* = 8) and patients suffering from HCMV^+^/cHBV- (*n* = 4) and HCMV^+^/cHCV infection (*n* = 11). The intracellular staining for FcεRIγ was not compatible with the staining protocol for phosphorylated STAT proteins. CD56^dim^ NK cell clusters were separated by the expression of the surface markers CD2, CD57 and CD85j (see Figure S5C). Bar charts indicate the median value with IQR. MFI of ISG expression was normalized to CD3^−^CD56^+^ NK cells. Statistical analyses were performed via (A) Kruskal-Wallis test and (B) Friedman test, ∗: *p* < 0.05, ∗∗: *p* < 0.01, ∗∗∗: *p* < 0.001, ∗∗∗∗: *p* < 0.0001. IFNα, interferon-alpha; IFN, interferon. n.s., not significant. See also Figures S4–S7.

To assess how differences in the inflammatory response may affect ISG signatures in NK cells, we investigated HCMV^+^ chronic HCV and HBV infections (type-I/III IFN-dominated versus -neglected) as model systems to compare ISG profiles. Four NK cell clusters (CD56^bright^, cCD56^dim^, and adCD56^dim^ I and II) were found in chronic HBV and HCV infection, similar to HD (Figures 2A, 2B, and S3A). In addition, all previously defined overall ISG patterns were mostly conserved at the transcriptome and protein level among the different cohorts; however, with partially considerable data variations in particular within CD56^bright^ NK cells (Figures 2C–2E and S3B). Similar to the HD cohort, IFITM3 and IRF1 were highly expressed in CD56^bright^ NK cells and diminished in cCD56^dim^ and/or adCD56^dim^ NK cells of chronically HBV- or HCV-infected patients. Whereas, IFIT2 and ISG20 were reduced in CD56^bright^ NK cells compared to cCD56^dim^ NK cells (Figures 2D, S3C, and S3D). According to the constant NK cell subset distribution during the course of chronic infection with HBV or HCV (Figure S4A), expression levels of IFITM3, IRF1 as well as IFIT2 and ISG20 remained stable, being even independent of NUC or DAA treatment (Figures S4B and S4C). These findings suggested a potentially limited regulation of these ISGs by IFN signaling. A limited IFN signaling was supported by gene set enrichment analysis using the Gene Ontology (GO) term IFN-mediated signaling pathways (Figure S4D). To investigate the effect of IFNs on ISG expression in NK cells in more detail, we performed *in vitro* stimulation. Of note, ISG expression levels in NK cells were partly different in this experimental *in vitro* setting compared to *ex vivo* analyses. Thus, we strictly compared the ISG expression of NK cell subsets after IFN stimulation with mock stimulation. We did not observe any increased expression levels of IFITM3, IRF1, IFIT2, and ISG20 upon type-I (IFNα2, IFNβ), -II (IFNγ) and -III (IFNλ1, IFNλ3) IFN stimulation in NK cells from HD and patients with chronic viral infections (Figures 3A, S4E, and S5A). Notably, the gene expression of *IFNAR1, IFNGR1* but not *IFNLR1* (Figure S5B) and protein expression of IFNAR2 (Figure S5C) were detectable in NK cell clusters from HD and patients suffering from chronic HBV or HCV infection and STAT1 and/or STAT4 were phosphorylated upon type-I and -II IFN stimulation (Figures 3B, S5D, and S5E). Thus, NK cells were responsive to type-I and -II but not -III IFN stimulation; however, the expression of distinct ISGs was not increased in this experimental *in vitro* setting. Moreover, ISG expression of the respective NK cell subsets did also not correlate with the clinical parameters ALT, AST and viral load (Figures S6A–S6D and S7A–S7D). Combined, these data may indicate that the expression of distinct ISGs is not guided by IFN signaling and conserved in NK cells irrespective of the presence of a chronic viral infection.

### Association of interferon-stimulated gene expression and natural killer cell differentiation

**Figure 4:**
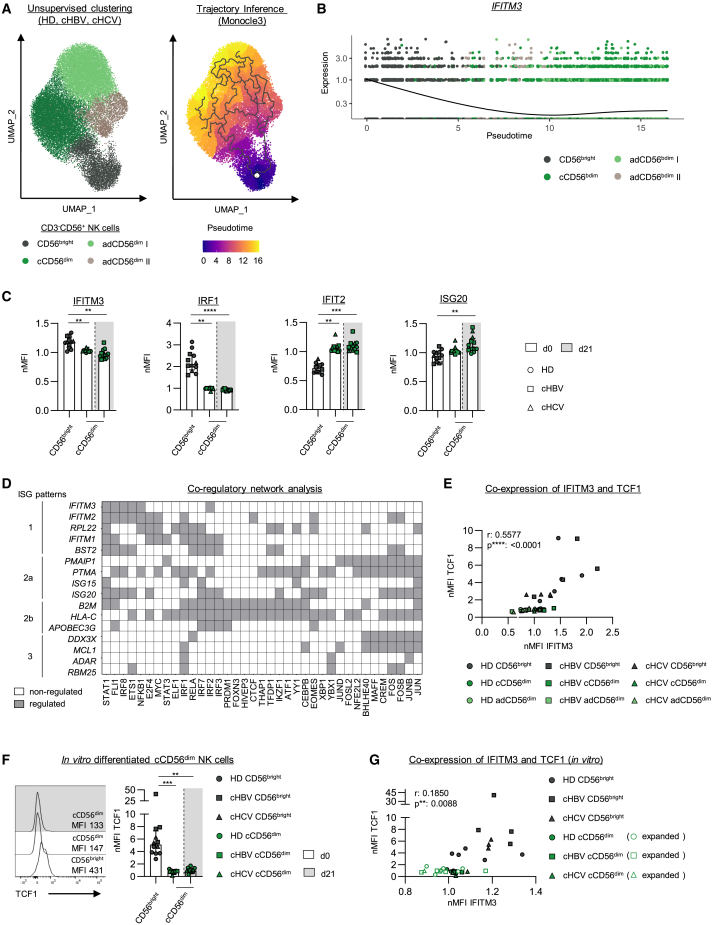
ISG expression is associated with NK cell differentiation regulated by transcription factor networks (A) Merged unsupervised clustering of NK cell clusters (dark gray: CD56^bright^, green: cCD56^dim^, light green: adCD56^dim^ I and beige: adCD56^dim^ II) and trajectory inference analysis of NK cell clusters merged from HCMV^+^/HD, HCMV^+^/cHBV- and HCMV^+^/cHCV-infected patients. Each dot represents one CD3^−^CD56^+^ NK cell. (B) *IFITM3* expression profile with respect to pseudotime of the different NK cell clusters. The line indicates the fitting of the expression trend over pseudotime. (C) Statistical graphs depicting ISG expression in CD56^bright^ and cCD56^dim^ NK cells of HCMV^+^/HD (*n* = 5), HCMV^+^/cHBV- (*n* = 4) and HCMV^+^/cHCV-infected patients (*n* = 3) *ex vivo* (white) and *in vitro* after differentiation at day 21 (light gray) measured by flow cytometry. (D) Binary heatmap represents the co-regulatory network analysis of the previously introduced four ISG sets and distinct transcription factors. White indicates non-regulation and gray indicates regulation. (E) Co-expression analysis of TCF1 and IFITM3 on protein level in CD56^bright^, cCD56^dim^, and adCD56^dim^ NK cells from HCMV^+^/HD (*n* = 4), HCMV^+^/cHBV- (*n* = 4) and HCMV^+^/cHCV-infected patients (*n* = 5) is shown. (F and G) Representative flow cytometry dot plot and bar chart with percentage of (F) TCF1 expression and (G) co-expression analysis of TCF1 and IFITM3 of *in vitro* differentiated cCD56^dim^ NK cells (light gray) compared to *ex vivo* ISG expression in CD56^bright^ and cCD56^dim^ NK cells (white) from HCMV^+^/HD (*n* = 5), HCMV^+^/cHBV- (*n* = 4) and HCMV^+^/cHCV-infected patients (*n* = 3). Bar charts indicate the median value with IQR. MFI of ISG expression was normalized to CD3^−^CD56^+^ NK cells. Statistical significance was assessed by (D and G) one-way repeated measures ANOVA (IFITM3), Friedman test (IRF1, ISG20, IFIT2, and TCF1), and (F and H) simple linear regression. ∗: *p* < 0.05, ∗∗: *p* < 0.01, ∗∗∗: *p* < 0.001, ∗∗∗∗: *p* < 0.0001. See also Figure S8.

Next, we tested whether ISG expression in HD and chronic viral hepatitis is linked to certain NK cell states. For this, we performed trajectory inference of the different NK cell clusters from the scRNAseq data (Figure 4A) to assess the relationship of these identified cell states and thus differentiation paths. Trajectory analysis indicated that CD56^bright^ NK cells give rise to cCD56^dim^ and adCD56^dim^ I and II NK cells and thus suggested a progenitor/progeny relationship of these cells. ISG expression (e.g., *IFITM3*) dynamically changed along the trajectory, supporting an association of ISG expression and NK cell differentiation (Figure 4B). To further validate and corroborate this hypothesis, we performed *in vitro* NK cell differentiation assays.^18^^,^^19^ For this, we purified CD56^bright^ NK cells from HD and chronically HBV- or HCV-infected patients by FACS sorting and subsequently expanded these cells with Interleukin-2 (IL-2) over 21 days. We observed the emergence of cCD56^dim^ phenotype NK cells by the upregulation of CD16 and downregulation of CD56 (Figures S8A and S8B). E*x vivo* ISG expression of CD56^bright^ and cCD56^dim^ NK cells were compared with the ISG expression of expanded cCD56^dim^ NK cells (Figures 4C and S8C–S8E). Indeed, *ex vivo* IFITM3 expression was higher in CD56^bright^ NK cells compared to cCD56^dim^ NK cells (Figure 2D). Strikingly, *in vitro* differentiation was linked to a reduction of IFITM3 expression, and subsequently, *in vitro* differentiated cCD56^dim^ NK cells showed similar IFITM3 expression to the *ex vivo* IFITM3 expression level in cCD56^dim^ NK cells. In addition, we observed that IRF1 expression was also reduced during the *in vitro* differentiation of NK cells, while IFIT2 and ISG20 expression were upregulated. The expression of both ISGs, IFIT2 and ISG20, was increased in cCD56^dim^ NK cells compared to CD56^bright^ NK cells, and *in vitro* differentiated and *ex vivo* cCD56^dim^ NK cells showed similar expression of IFIT2 and ISG20. Thus, ISG expression changed according to the differentiation state of NK cells in all three tested cohorts, supporting that ISG expression can be linked to NK cell differentiation.

### Co-regulation of interferon-stimulated gene and transcription factors determining natural killer cell states

To identify transcription factors involved in ISG regulation, additionally to interferon stimulated gene factor 3 (ISGF3) or STAT1 homodimers, in NK cells, we conducted co-regulatory network analysis of the scRNAseq data. We grouped the respective ISGs according to the four previously defined ISG patterns to identify overlapping co-regulons of ISGs with transcription factors expressed in NK cells (Figures 4D and S8F). Binary matrix shows distinct transcription factors, which regulated specific ISGs (Figure 4D). With respect to ISG pattern 1 linked to CD56^bright^ NK cells, we identified overlapping co-regulation with, e.g., ETS1 and FLI1. Additionally, EOMES and PRDM1*,* for example, showed co-regulation with ISGs from patterns 2a and 2b linked to CD56^dim^ NK cells. These transcription factors are known to be associated with NK cell differentiation/maturation. Furthermore, ISGs included in pattern 3 were co-regulated with, e.g., CREM, FOS, and JUNB. Another important transcription factor of CD56^bright^ NK cells is TCF1, which we found to be co-expressed with IFITM3, a surrogate marker ISG of pattern 1, in CD56^bright^ NK cells (Figure 4E). In contrast, in cCD56^dim^ and adCD56^dim^ NK cells, both, TCF1 and IFITM3, were downregulated. This dynamic co-expression of IFITM3/TCF1 was also recapitulated during *in vitro* NK cell differentiation from CD56^bright^ to cCD56^dim^ NK cells (Figures 4F and 4G). Hence, the regulation of ISG in NK cells was associated with transcription factors, which are involved in NK cell differentiation.

### Interferon-stimulated genes are linked to general biological processes in natural killer cells' anti-viral activity

**Figure 5:**
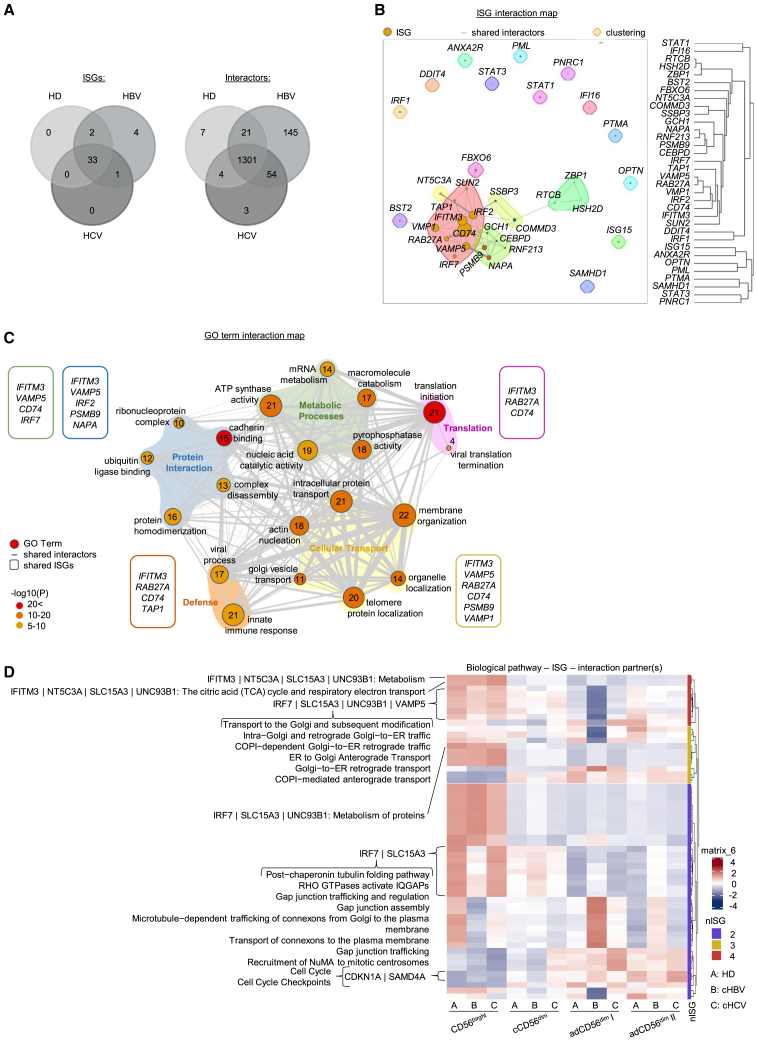
ISG sets in NK cells linked to biological pathways Comparative protein-interaction networks of ISGs expressed in total CD3^−^CD56^+^ NK cells from HCMV^+^/HD and patients suffering from HCMV^+^/cHBV and HCMV^+^/cHCV with cellular proteins (based on Hubel et al., Nat Immunol. 2019). (A) Overlap analysis of expressed ISGs and interactors between patient groups. (B) ISG interaction map showing the distinct ISGs with one or more interactions with cellular proteins. Node size indicates the number of interactors, and edge width shows the number of shared interactions. (C) GO term interaction map depicting biological processes in which distinct ISGs are involved. Each node indicates a specific pathway, and the size of the node represents the number of involved ISG interactors that are expressed in NK cells. The number of shared ISG-interacting cellular proteins between two pathways is indicated by the thickness of the connecting line. Colored background marks group distinct pathways together in one general category. Involved shared ISGs of one general category are depicted in the colored squares. (D) Heatmap depicting biological pathway expression in NK cell clusters that are co-regulated by distinct, co-expressed ISGs. nISG: number of ISG.

Next, we investigated whether ISGs are involved in biological processes beyond the anti-viral state in NK cells in patients with HD and chronic viral hepatitis. For this, we aligned the protein-interaction network of ISGs with cellular proteins as reported by Hubel et al. with our scRNAseq data of NK cells.^14^ In total, we identified 33 ISGs and 1301 interactors with overlapping expression in NK cells from HD and similarly in patients with chronic HBV and HCV infection (Figure 5A). Out of the 33 ISGs, 55% exhibited co-expression with one or more interacting cellular proteins in NK cells (Figure 5B). In total, we were able to detect 434 potential interactions between 28 ISGs and 359 cellular proteins, of which approximately 85% were related to general biological processes according to GO enrichment analysis (Figure 5C). These identified ISG-involving biological processes in NK cells could be grouped into five general categories: metabolic processes, cellular transport, protein interaction, translation, and defense. Of note, IFITM3 was implicated in all biological processes within the five categories. Cellular transport, metabolic processes, translation, and defense shared the ISG CD74. In addition, VAMP5 and RAB27A were involved in all processes of the three general categories. However, the combinatorial expression of IFITM3, PSMB9, and VMP1, for example, associated with BAG5, NOLC1, or RAB18 being involved in transport pathways, and IFITM3, CD74, and VAMP5 are linked to metabolic processes via ATF4, EIF3E, or VAPA. Taking combinatorial ISG expression into account (Figure 5D), the co-expression of ISGs and known interaction partners involved in the regulation of metabolic activity and ER-Golgi transport was most prominent in CD56^bright^ NK cells. Furthermore, in cCD56^dim^ NK cells expression of ISGs and interactors within gap junction pathways was linked, and finally, we also detected the co-expression of cell cycle control proteins and ISGs, particularly in adaptive NK cells. In sum, these data reveal that in NK cells ISGs are potentially also involved in cellular transport and metabolism, in addition to anti-viral defense pathways.

## Discussion

With this study, we extend our view on ISGs in NK cell biology beyond mediating a direct interferon-induced anti-viral state, and by this, highlight versatile roles of ISGs in the immune response. In particular, we identified conserved ISG expression patterns in circulating NK cells from HD, chronically HBV- and HCV-infected patients. Since in HCV infection, a strong IFN response is induced, contrary to infection with HBV, a so-called “stealth” virus,^17^^,^^20^^,^^21^ these similar ISG patterns in NK cells from HD, HBV- and HCV-infected patients potentially indicate IFN-independent expression. An IFN-independent expression is further supported by the fact of similar ISG expression in NK cells of healthy donors without clinical signs or reports of inflammatory processes or infections. This observation of NK cells expressing high levels of ISGs in healthy donors is in line with previous reports of so-called “inflamed” NK cell populations. These populations exhibit an increased ISG expression and were found in the blood and in the bone marrow of HD without a recent history of immune action.^22^^,^^23^ It cannot be ruled out that autocrine IFN signaling, especially by IFNγ, contributes to the observed expression of ISGs in NK cells. Yet, we rather observed a significantly increased ISG expression in CD56^bright^ NK cells that is linked to the NK cell differentiation state. Trajectory analysis of NK cells and *in vitro* NK cell differentiation further revealed that the expression of certain ISGs is down-regulated following the progenitor/progeny differentiation of CD56^bright^ to CD56^dim^ NK cells. This ISG expression pattern was observed irrespective of whether the NK cells were obtained from HD or patients suffering from chronic viral hepatitis. Furthermore, it is reminiscent of the reported IFN-independent, intrinsic ISG expression in hESC that decreases with differentiation.^15^^,^^16^

In line with a constitutive ISG expression in NK cells, we also showed that the expression of IFITM3, IRF1, IFIT2 and ISG20 are refractory to IFN stimulation *in vitro* in NK cells that were obtained from patients with HD and chronic viral hepatitis. Indeed, while NK cells can be activated by IFNs via phosphorylated STAT molecules to elicit effector function,^24^ parallel analysis of ISG expression demonstrated limited up-regulation upon IFN stimulation. Possible mechanisms underlying sensitivity versus refractoriness of NK cells to IFNs with respect to effector function and ISG induction, respectively, may be abrogated formation of transcription factor complexes such as ISGF3^25^ or epigenetic silencing of IRFs (interferon response elements).^26^ Future studies are, however, required to unravel the mechanistic underpinnings. Still, our findings are again in accordance with the previous observation made with regard to hESCs that are also unable to activate the full range of ISGs upon type-I IFN stimulation^16^ and thus exhibiting a distinct refractoriness of ISG induction by type-I IFNs. Refractoriness of stem cells toward type-I IFNs may be beneficial due to their known anti-proliferative activity mediated by a specific group of ISGs. This anti-proliferative activity would contradict the role of stem cells in tissue regeneration, repair, and self-renewal.^27^ Thus, with regard to NK cells, anti-proliferative activity would counteract the rapid activation and expansion when damage signals appear, type-I IFNs are subsequently induced, and an immune response needs to be established. Simultaneously, not all NK cells must be recruited to the most differentiated effector cell state after activation and potential boosting by IFNs. Hence, ISG refractoriness accompanied by the constitutive expression of ISGs may represent a rheostat to regulate NK cell expansion and accompanied differentiation in HD as well as during chronic viral hepatitis.

Regulon analysis additionally highlighted the co-regulation of several ISGs by IFN-independent transcription factors including transcription factors that have already been linked to NK cell maturation, differentiation and function. For example, FLI1, involved in the formation of memory precursor NK cells,^28^ MYC that is important for immature NK cells with high proliferative capacity^29^^,^^30^ and ETS1, a key factor in early NK cell development^31^ were co-regulated with ISGs that were highly expressed in CD56^bright^ NK cells. In addition, IFITM3 and TCF1 were co-expressed in CD56^bright^ in contrast to CD56^dim^ NK cells, according to the role of TCF1 as an essential key regulator in NK cell development that restricts terminal differentiation.^29^ Moreover, we found Blimp-1 and Eomes to be linked to ISGs highly expressed in further differentiated CD56^dim^ NK cells. Eomes plays an important role in the maturation, differentiation, and functionality of NK cells,^32^^,^^33^ and Blimp-1 has been reported to be increasingly expressed during NK cell differentiation and to control NK cell function.^34^^,^^35^ Hence, our data clearly suggest that distinct ISGs are co-regulated with fate-determining transcription factors of NK cells and therefore rather associated with differentiation states, probably uncoupled from IFN-dependent conditions.

According to the role of intrinsic ISG expression in hESC protecting from viral infections^15^^,^^16^ it is tempting to speculate that constitutive ISG expression mediates a similarly protected state of NK cells, especially of the CD56^bright^ cluster. Hence, besides the stem cell niche, a niche-protecting function of constitutive ISG expression may also be evident within the immune system. This anti-viral activity might be on the one hand, necessary to protect from invading viruses, but on the other hand, also from endogenous retroviruses. Indeed, several viruses can potentially infect NK cells, including EBV, HSV, VZV, or VV, especially via cell-cell interactions with infected cells, endocytosis/micropinocytosis, or entry receptor acquisition upon trogocytosis.^36^ Furthermore, approximately 8% of the human genome is composed of transcriptionally inactive retroviral DNA from human endogenous retroviruses (HERV) that are frequently activated in inflammatory conditions and during viral infections.^37^^,^^38^ This reactivation requires an endogenous safeguard immune mechanism for control that is potentially of special importance in chronic viral infections such as those with HBV or HCV due to persisting inflammatory processes. However, the relevance of constitutive ISG expression in NK cells may go beyond the establishment of an anti-viral state. In a mass spec-based protein-protein interaction screen, Hubel et al. identified a role of ISGs in cellular pathways such as transport, metabolism, and gene expression beyond anti-viral defense.^14^ Integration of our data with known cellular interaction partners of ISGs identified by Hubel et al. clearly indicate a role of ISGs in general biological processes such as cellular transport, metabolism, mRNA translation, and cell cycle control in NK cells. In particular, in CD56^bright^ NK cells, the co-expression of ISGs and known interaction partners involved in the regulation of metabolic activity and ER-Golgi transport was evident. CD56^bright^ NK cells have been reported to exhibit a higher metabolic activity and ER-Golgi transport capacity in order to more rapidly mobilize IFNγ.^39^^,^^40^^,^^41^ In addition, in cCD56^dim^ NK cells, the expression of ISGs and interactors within gap junction pathways was connected. Gap junctions are important for intercellular communication and, by this, regulate NK cell activation and cytotoxic activity.^42^ Finally, in line with the reported association of proliferation and adaptive NK cell phenotype,^43^ we detected the linked expression of cell cycle control proteins and ISGs in adaptive NK cells. Hence, these data support that ISG expression in NK cells is associated with key characteristics of the respective NK cell subset.

The observation that sets of ISGs were linked to NK cell subsets rather than single ISGs suggested combined action. Combined action of ISGs organized in networks has already been described before in the context of host defense, antiproliferative activities, epigenetic modulation, and adaptive immune stimulation.^3^^,^^44^^,^^45^ Hence, we focused on the analysis of ISG networks within NK cells and therefore did not address the specific and causal role of distinct ISGs such as IFITM3, IRF1, IFIT2, and ISG20 on NK cell function and differentiation. Accordingly, we identified biological pathways rather than single interaction partners or modulators linked to the expression of the respective ISG sets in NK cells. Another limitation of our study is the inclusion strategy for HBV-infected patients restricted to chronic infection with low viral load and low transaminases. Although this is the most common patient group in advanced countries, it does not represent the full heterogeneity of chronic HBV infection, known to be characterized by different NK cell alterations and inflammatory responses. Nevertheless, in sum, here we showed that the expression of ISG networks in NK cells goes beyond the establishment of an IFN-dependent anti-viral state, consequently revealing broad and versatile functions of ISGs within the immune system in health and disease.

### Limitations of the study

We concentrated on analyzing ISG networks in NK cells and, as a result, did not explore the specific or causal roles of individual ISGs such as IFITM3, IRF1, IFIT2, and ISG20 in NK cell function and differentiation. Consequently, our findings highlight broader biological pathways rather than pinpointing specific interaction partners or modulators associated with these ISG sets in NK cells. Another limitation of our study lies in the selection criteria for HBV-infected patients, which focused on individuals with chronic infection, low viral load, and low transaminase levels. While this subgroup reflects the most common clinical presentation in developed countries, it does not capture the full diversity of chronic HBV infection, which includes a range of NK cell alterations and inflammatory profiles. In addition, the absence of patient data on race, ancestry, or ethnicity prevented us from considering potential influences of these variables in our analysis.

## Resource availability

### Lead contact

Requests for further information and resources should be directed to and will be fulfilled by the lead contact, Maike Hofmann (maike.hofmann@uniklinik-freiburg.de).

### Materials availability

This study did not generate new unique reagents. Biological material is not available because the small amount available to us for the experiments has been entirely used up.

### Data and code availability

• The scRNAseq data generated in this study have been deposited in the European Genome–Phenome Archive under the accession number EGA: EGAS00001007771. The data are available under controlled access due to the sensitive nature of sequencing data. Access can be obtained by contacting the data access committee listed for the dataset in the study. Access will be granted to commercial and non-commercial parties according to patient consent forms and data transfer agreements. We have an institutional process in place to deal with requests for data transfer. A response to requests for data access can be expected within 14 days. After access has been granted, the data is available for 2 years.
• This article does not report original code.
• Any additional information required to reanalyze the data reported in this article is available from the lead contact upon request.

## Acknowledgments

We thank all patients and volunteers for participating in the current study and the FREEZE-Biobank Center (Freiburg University Medical Center) for support. This study was supported by CRC/TRR179 Project 20 (to M.H.), CRC/TRR179 Project 01 (to R.T.), CRC/TRR179 Project 04 (to T.B.), CRC/TRR179 Project 21 (to B.B), CRC/TRR179 Project 02 (to C.N.H.), CRC/TRR179 Project 17 (to V.L.), CRC/TRR179 Project 11 (to M.B. and A.P.) and CRC1479/Oncoescape Project 10 (to M.H.) of the German Reasearch Foundation (10.13039/501100001659DFG; TRR179 project no. 272983813 and CRC1479/Oncoescape project no. 441891347). M.H. is supported by the 10.13039/501100001659DFG Heisenberg program (project no. HO 5836/2-1).

## Author contributions

F.K. designed, performed and analyzed all experiments. T.T., R.L.C., F.K. and S. performed the computational analysis of single cell RNA sequencing data. K.J., O.S., F.B. and L.B. contributed to the experiments. G.R recruited patients. T.B., B.B., C.N.H., M.R., R.E., A.P., M.B., V.L. and R.T. contributed to data interpretation. M.H. designed and supervised the study, contributed to experimental design, planning and data interpretation. M.H. and R.T. wrote together with F.K. the article.

## Declaration of interests

The authors of this study declare that they do not have any conflict of interest.

## STAR★Methods

### Key resources table

**Table:** 

REAGENT or RESOURCE	SOURCE	IDENTIFIER
**Antibodies**		

Mouse monoclonal anti-human CD2 (clone RPA-2.10)	BD Bioscience	Cat#740960; RRID:AB_2740585
Mouse monoclonal anti-human CD16 (clone 3G8)	BD Bioscience	Cat#561393; RRID:AB_10611579
Mouse monoclonal anti-human CD85j (clone GHI/75)	BD Bioscience	Cat#555942; RRID:AB_396239
Mouse monoclonal anti-human IRF-1 (clone 20/IRF-1)	BD Bioscience	Cat#566322; RRID:AB_2739684
Mouse monoclonal anti-human PLZF (clone R17-809)	BD Bioscience	Cat#564850; RRID:AB_2738984
pSTAT1 (pY701) (clone 4a)	BD Bioscience	Cat#562069; RRID:AB_11151907
pSTAT4 (pY693) (clone 38/p-Stat4)	BD Bioscience	Cat#562073; RRID:AB_10895804
Mouse monoclonal anti-human CD3 (clone SK7)	BioLegend	Cat#344822; RRID:AB_2563420
Mouse monoclonal anti-human CD56 (clone B159)	BioLegend	Cat#318318; RRID:AB_604107
Mouse monoclonal anti-human CD56 (clone 5.1H11)	BioLegend	Cat#362532; RRID:AB_2565602
Mouse monoclonal anti-human TCF-1 (clone 7F11A10)	BioLegend	Cat#655204; RRID:AB_2566620
Mouse monoclonal anti-human CD3 (clone SK7)	eBioscience	Cat#45-0036-42; RRID:AB_1518742
Mouse monoclonal anti-human CD14 (clone 61D3)	eBioscience	Cat#47-0149-42; RRID:AB_1834358
Mouse monoclonal anti-human CD19 (clone HIB19)	eBioscience	Cat#47-0199-42; RRID:AB_1582230
Mouse monoclonal anti-human CD57 (clone TB01)	eBioscience	Cat#47-0199-42; RRID:AB_1582230
Armenian hamster monoclonal anti-human Helios (clone 22F6)	eBioscience	Cat#48-9883-42; RRID:AB_2574136
Mouse monoclonal anti-human IFIT2 (clone F-12)	Santa Cruz Biotechnology	Cat#sc-390724 AF594
Mouse monoclonal anti-human ISG20 (clone D-5)	Santa Cruz Biotechnology	Cat#sc-376665 AF680
Rabbit anti-human FceRIg (polyclonal)	Milipore	Cat#FCABS400F; RRID:AB_11203492
Mouse monoclonal anti-human NKG2C (clone 134591)	R&D Systems	Cat#FAB138A-100
Mouse monoclonal anti-human INFAR-II (clone REA124/MMHAR-2)	Miltenyi Biotech	Cat#130-128-948; RRID:AB_3663811
Rabbit recombinant monoclonal anti-human IFITM3 (clone JU73-02)	Invitrogen	Cat#MA5-32798; RRID:AB_2810074

**Biological samples**		

EDTA anti-coagulated blood and serum samples from HCMV^+^ health donors	FREEZE Biobank Center - University of Freiburg, Germany	N/A
EDTA anti-coagulated blood and serum samples from HCMV^+^/cHBV-infected patients	FREEZE Biobank Center - University of Freiburg, Germany	N/A
EDTA anti-coagulated blood and serum samples from HCMV^+^/cHCV-infected patients	FREEZE Biobank Center - University of Freiburg, Germany	N/A

**Chemicals, peptides, and recombinant proteins**		

Human Recombinant IFN-alpha 2A	STEMCELL Technology	Cat#78076.1; conc: 1000IU/ml
Human Recombinant IFN-beta	STEMCELL Technology	Cat#78113.1; conc: 100 μg/ml
Human Recombinant IFN-gamma	STEMCELL Technology	Cat#78020; conc: 100 μg/ml
Recombinant Human IFN-λ1	STEMCELL Technology	Cat#711204; conc: 100 μg/ml
Recombinant Human IL-28A (IFN-λ3)	STEMCELL Technology	Cat#754202; conc: 100 μg/ml
Human Recombinant IL-2	STEMCELL Technology	Cat#78036.3; conc: 100UI/ml
Streptavidin	BD Bioscience	Cat#563262; RRID:AB_2869478

**Critical commercial assays**		

BD Phosflow™ Fix Buffer I	BD Bioscience	Cat#557870
BD Phosflow™ Perm Buffer III	BD Bioscience	Cat#558050
NK cell isolation Kit	Miltenyi Biotech	Cat#130-092-657
One-Step Antibody Biotinylation Kit	Miltenyi Biotech	Cat#130-093-385
Foxp3/Transcription 442 Factor Staining Buffer	eBioscience	Cat#00-5523-00
Chromium Single Cell 3′ Library & Gel Beads Kit v2	10x Genomics	N/A

**Deposited data**		

Raw and analyzed data	This paper	EGAS00001007771

**Software and algorithms**		

FlowJo	BD	https://www.flowjo.com/
GraphPad Prism V.9	GraphPad	https://www.graphpad.com/
CellRanger 489 software version 2.1.1	10x Genomics	RRID:SCR_017344
R Studio	R-Tools Technology Inc.	https://www.r-studio.com/de/
R package: SCENIC	R-Tools Technology Inc.	RRID:SCR_017247
R package: Seurat version 3.1.3	R-Tools Technology Inc.	RRID:SCR_016341

**Other**		

Monocle3 v0.2.0	Cao et al.^46^	N/A
ISG gene sets	Wu et al.^16^	N/A
ISGs and their interactors	Hubel et al.^14^	N/A

### Experimental model and study participant details

#### Study cohort

EDTA anti-coagulated blood and serum samples from healthy donors (*n* = 18) and patients infected with hepatitis B (*n* = 19) and hepatitis C virus (*n* = 20) were obtained at the Department of Medicine II of the University Hospital Freiburg. For detailed patient information such as sex, age, genotype, therapy status and clinical parameters see Tables S1–S3. Prior blood donation written informed consent was collected from all healthy donors and patients. The study was conducted according to federal guidelines, local ethics committee regulations of Albert-Ludwigs-Universität, Freiburg, Germany (no. 474/14, 383/19, 515/19) and the Declaration of Helsinki (1975). Based on the guidelines and ethical regulations, information on race, ancestry, or ethnicity of human participants was not collected.

### Method details

#### PBMC isolation

Isolation of peripheral blood mononuclear cells (PBMCs) from EDTA anti-coagulated blood samples was performed by density gradient centrifugation using Pancoll (Pan-Biotech, Germany). For all analyses, cryopreserved PBMCs were thawed in IMDM culture medium (IMDM, 10% fetal bovine serum, 1% penicillin-streptomycin (all Thermo Fischer Scientific, Waltham, MA, USA) and 50 μM β-mercaptoethanol (Sigma-Aldrich, St. Louis, MO, USA)).

#### HCMV status

Human cytomegalovirus (HCMV) serostatus from all healthy donors and patients were determined by the Department of Virology, University Freiburg through HCMV-IgG Chemiluminescence Immunoassay (DiaSorin LIAISON).

#### Multiparametric flow cytometry

For multiparametric flow cytometry the following monoclonal and polyclonal antibodies were used: anti-CD2 (RPA-2.10), anti-CD16 (3G8), anti-CD85j (GHI/75), anti-IRF1 (20/IRF-1), anti-PLZF (R17-809) (BD Bioscience, Franklin Lakes, NJ, USA), anti-CD3 (SK7), anti-CD56 (B159 and 5.1H11), anti-TCF1 (7F11A10) (BioLegend, Sant Diego, CA, USA), anti-CD3 (SK7), anti-CD14 (61D3), anti-CD19 (HIB19), anti-CD57 (TB01), anti-Helios (22F6) (eBioscience, San Diego, CA, USA), anti-IFIT2 (F-12), anti-ISG20 (D-5) (Santa Cruz Biotechnology, Dallas, TX, USA), anti-FceRIg (polyclonal) (Milipore, Billercia, MA, USA), anti-NKG2C (134591) (R&D Systems, Minneapolis, MN, USA), anti-INFAR-II (REA124/MMHAR-2) (Miltenyi Biotech, Germany).

For live/dead discrimination of the cells Fixable Viability Dye (eFlour780, eBioscience, Germany) was used. Foxp3/Transcription Factor Staining Buffer (eBioscience, Germany) was used according to the manufacturer's instruction for intracellular and intranuclear staining. For measuring on FACSCanto II and LSRFortessa (BD Bioscience) the cells were fixed with 2% paraformaldehyde. Data analysis were performed via FlowJo software (Treestar, Ashland, OR, USA).

#### Detection of phosphorylated STAT proteins

For detection of phosphorylated STAT proteins (pSTAT1 (pY701, 4a) and pSTAT4 (pY693, 38/p-Stat4 (BD Bioscience, Franklin Lakes, NJ, USA)), 1x10^6^ PBCMs were plated in V-bottom 96 well plates in IMDM culture medium and incubated for 2 h. PBMCs were stimulated with Interferon-alpha 2A (IFNα, STEMCELL Technology, Vancouver, Kanada) with a final concentration of c = 1000IU/ml or without (as negative control) for 3 h. Afterwards surface-antibodies were stained for 10min. Directly fixation was performed with one volume pre-warmed 37°C BD Phosflow™ Fix Buffer I (BD Bioscience, Germany) for 10 min. All steps were incubated at 37°C and 5% CO_2_. After washing twice with staining buffer (SB, 1x D-PBS, 1% fetal bovine serum) PBMCs were permeabilized with pre-cool −20°C BD Phosflow™ Perm Buffer III (BD Bioscience, Germany) and were incubated for 30 min at 4°C, in the dark. After three-time washing with SB, PBMCs were stained with phospho-antibodies for 60 min at room temperature (RT), in the dark. After washing with SB the PCMBs were measured by flow cytometry.

#### Detection of IFITM3 protein

Pure anti-IFITM3 antibody (JU73-02, Invitrogen, Waltham, MA, USA), was biotinylated with the One-Step Antibody Biotinylation Kit (Miltenyi Biotech, Germany) according to the manufacturer's instruction. All antibody stainings that included anti-IFITM3 biotinylated antibody in the intracellular/intranuclear staining (Foxp3/Transcription Factor Staining Buffer (eBioscience)) were divided in two steps. In step 1 only anti-IFITM3 biotinylated antibody was added to PBMCs and was incubated for 30 min at 4°C in the dark. After washing twice, PBMCs were incubated in step 2 with all other intracellular/intranuclear antibodies and Streptavidin (BV711, BD Bioscience, Germany) (for detection of anti-IFITM3 biotinylated antibody) for 30 min at 4°C in the dark.

#### IFNα stimulation

IFNα stimulation was performed by seeding 1x10^6^ PBMCs in V-bottom 96 well plates in IMDM culture medium. PBMCs were incubated 14–16 h in the presence of IFN2α (STEMCELL Technology; 1000IU/ml), IFNβ1 (STEMCELL Technology; 100 μg/ml), IFNγ (STEMCELL Technology; 100 μg/ml), IFNλ1 (BioLegend; 100 μg/ml) IFNλ3 (BioLegend; 100 μg/ml), or with medium alone (as negative control) at 37°C and 5% CO_2_. Intracellular and intranuclear staining was performed with Foxp3/Transcription Factor Staining Buffer (eBioscience) as described before.

#### *In vitro* NK cell differentiation

PBMCs were stained with surface antibodies (anti-CD3, anti-CD14, anti-CD19 anti-CD16, anti-CD56 and Fixable Viability Dye). 10.000 CD56^bright^ NK cells (live, anti-CD3^-^/CD14^-^/CD19^-^ and anti-CD56^+^/CD16^-^) were sorted in U-bottom 96 well containing expansion medium (RPMI1640 (Thermo Fischer Scientific, Waltham, MA, USA), 1% penicillin-streptomycin, 10% human serum (Type AB, male, Pan-Biotech, Germany)). After finishing the sort plate were centrifuged for 4min, 1600 rpm, RT. Supernatant was removed and sorted CD56^bright^ NK cells were resuspended in 200 μL expansion medium containing 100UI/ml Interleukin-2 (rIL-2, 20 IU mL^−1^, Stemcell Technologies, Canada) and were incubated at 37°C and 5% CO_2_ for 20–22 days. Every 3–4 days, fresh expansion medium with a final concentration of 100UI/ml IL-2 was supplemented. PBMCs from healthy donors were sorted with FACSMelody Cell Sorter (Becton Dickinson). Sorting of PBMCs from cHBV- and cHCV-infected patients were performed with CytoFLEX SRT Brenchtop Cell Sorter (Beckman Coulter).

#### NK cell isolation

For negative NK cell isolation from PBMCs the human NK cell isolation Kit from Miltenyi Biotech, Germany was used. The isolation was done according to the manufacturer's instruction. Non-NK cells e.g., T cells, B cells, stem cells, dendritic cells, monocytes, granulocytes and erythroid cells are magnetically separated from untouched NK cells during this isolation.

#### Single-cell RNA sequencing via 10x Genomics single cell 3′ scRNA-seq technology

For scRNAseq 15.000 cells, negatively enriched for NK cells (corresponding to section NK cell isolation), from 2 HCMV^+^ healthy donors (HD1, HD2, corresponding to Tables S1–S3), 3 HCMV^+^ chronically HBV (HBV1, HBV2, HBV3), and 3 HCMV^+^ chronically HCV-infected patients (HCV1, HCV2, HCV3), each, were used as input. For GEM Generation & Barcoding, POST GEM-RT Cleanup & cDNA Amplification and Library Construction the Chromium Single Cell 3′ Library & Gel Beads Kit v2 was used according to the manufacturer's instruction. Raw sequencing reads for single cell transcriptome libraries were aligned using the CellRanger software version 2.1.1 (10x Genomics; RRID:SCR_017344), together with the human reference genome hg19. Low quality cells were removed using Seurat version 3.1.3 (RRID:SCR_016341)^47^ based on the number of detected genes (<200) and RNA molecules (>6000), as well as percentage of mitochondrial reads (>10%). Cells passing this quality control step were further processed using the Seurat software. The data was log-normalized to account for library size and scaled with mean centering by sample. Samples were then integrated by batch using canonical correlation analysis (CCA) and mutual nearest neighbor (MNN) matching as implemented in Seurat (“IntegrateData” function) using 50 principal components. Dimensionality reduction was performed on the integrated data by PCA using the 2000 most variable genes. Cells were clustered with the “FindClusters” function using 20 principal components for the neighborhood graph and the resolution parameter set to 0.4. Visualization was achieved by UMAP using the umap-learn algorithm as implemented in Seurat and 50 principal components.

#### Cell type assignment

Cell types were assigned using canonical marker gene expression. All cells not assigned to be NK cells (*NCAM*^*+*^*CD3E*^*-*^*CD3G*^*-*^*CD3D*^*-*^*CD19*^*−*^) were excluded from further analysis (1,897 cells; 5.2% of all cells that passed QC). For this study 14752 cells from HD1, 14959 cells from HD2, 9769 cells from HBV1, 9921 cells from HBV2, 10030 cells from HBV3, 9957 cells from HCV1, 9923 cells from HCV2 and 9831 cells from HCV3 were used for identifying of NK cell subpopulations, defined by a combination of NK cell marker gene expressions e.g., *NCAM*, *FCGR3A*, *FCER1G*, *KLRC2* and *ZBTB16*).

#### Trajectory inference

To infer the lineage relationship of the NK cell types, Monocle3 v0.2.0 was used.^46^ Trajectory graph learning and pseudo-time measurement through reversed graph embedding were performed.

#### Analysis of selected stem-like ISG signatures

Average expression of ISG gene sets defined by Wu et al.^16^ over all cells was calculated for each cell subpopulation as well as subpopulation split by the different study cohorts (HCMV^+^/HD, HCMV^+^/cHBV, HCMV^+^/cHCV) separately on the scaled expression data.

#### Analysis of ISG associated transcription factors

For the analysis of transcription factors and genes regulated by them, SCENIC (RRID:SCR_017247) was used. The relative proportion of respective ISGs associated with specific transcription factors across the four ISG sets was quantified.

#### Interferon-mediated signaling GO term expression

To evaluate the expression of the GO term “Interferon-mediated Signaling Pathway” (GO:0140888) in the NK cells of the scRNAseq data, we compiled the genes involved in this pathway and calculated a score using the AddModuleScore function of Seurat. This generates an average expression of the GO term that is subtracted by the aggregated expression of control genes in the scRNAseq dataset.

#### ISG interaction networks

Using the Hubel et al. study as the foundation for identifying ISGs and their interactors, we limited the analysis to genes that are expressed by at least 10% of all cells in our study. ISG interaction networks were generated with node size indicating the ISG’s number of interactors and the edge width shows the number of shared interactors between ISGs. Clustering of ISGs were based on their shared interactors to identify ISG neighborhoods with hierarchical clustering. To characterize the functional role of ISGs in our study, the interaction partners of each ISG were parsed through Metascape.^48^ A summary of enriched GO terms and the ISGs they’re associated with were generated by their known interactors from Hubel et al. Interaction networks were generated with the R package igraph v1.2.6.

### Quantification and statistical analysis

#### Statistics

Statistical analyses test were performed with GraphPad Prism V.9 (GraphPad Prism, USA). Graphical chars show the median values with IQR. Which statistical tests and sample size are used are noted in the figure legends. Levels of significance are specified as followed: ∗: *p* < 0.05; ∗∗: *p* < 0.01; ∗∗∗: *p* < 0.001; ∗∗∗∗: *p* < 0.0001.
