# Supplementary material for: Differentiation-associated ISG expression of NK cells in chronic viral infection
Source: iScience. 2025 Jul 26;28(9):113216. doi: 10.1016/j.isci.2025.113216 (PMC12362017; doi:10.1016/j.isci.2025.113216)
Supplement: Document S1. Figures S1–S8 and Tables S1–S3 [file mmc1.pdf]

iScience, Volume *28*

Supplemental information

Differentiation-associated ISG expression of NK cells in chronic viral infection

Franziska Keller, Robert Lorenz Chua, Timo Trefzer, Katharina Jechow, Liane Bauersfeld, Fabian Beier, Sagar, Özlem Sogukpinar, Giuseppe Rusignuolo, Marta Rizzi, Roland Eils, Andreas Pichlmair, Marco Binder, Bertram Bengsch, Christoph Neumann-Haefelin, Volker Lohmann, Tobias Boettler, Christian Conrad, Robert Thimme, and Maike Hofmann

Supplemental Figures

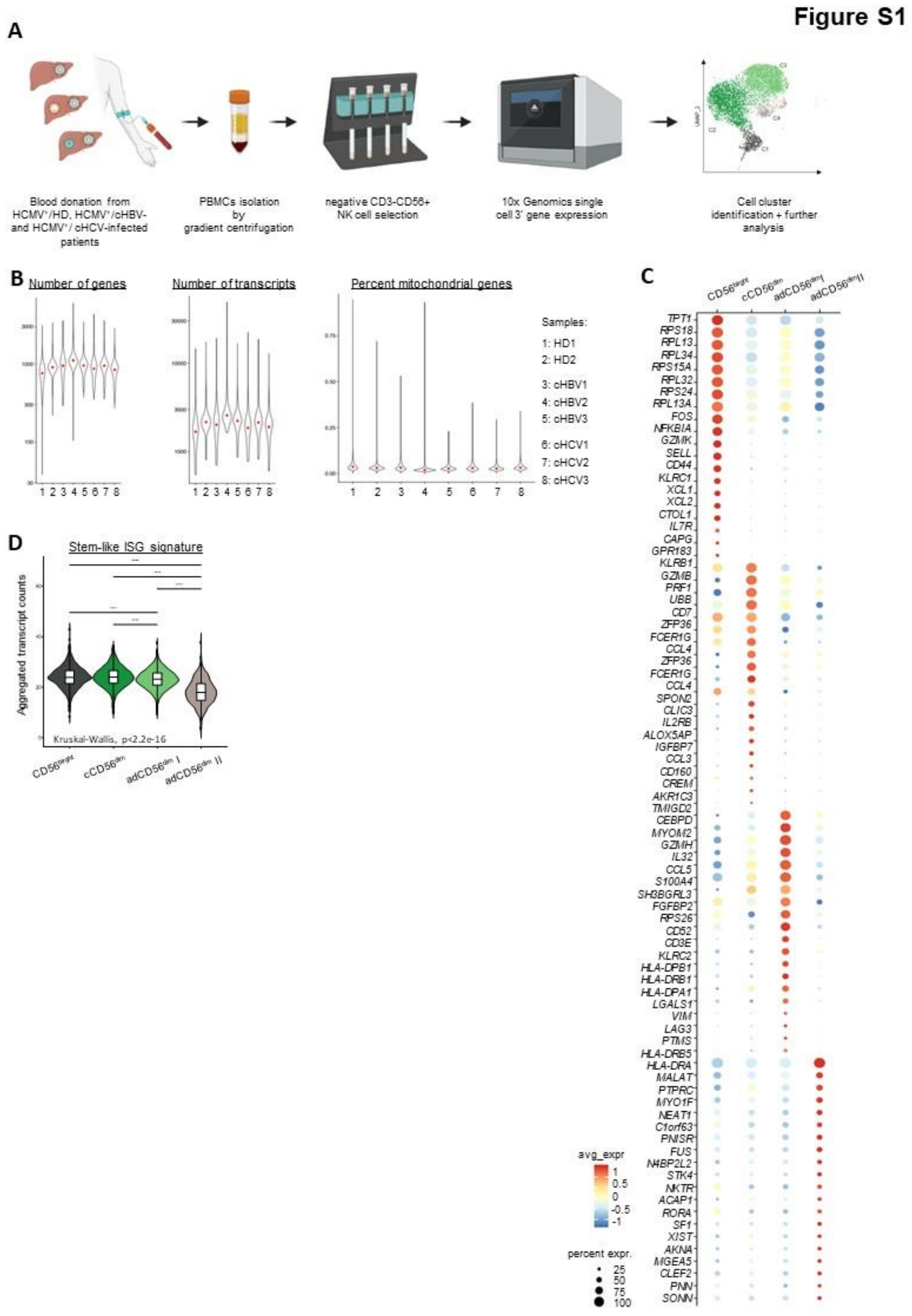

Figure S1: scRNAseq analysis of NK cells, <u>related to STAR Methods.</u> (A) Experimental setup for scRNAseq analysis via 10x Genomics technology is illustrated. **(B)** General metrics of scRNAseq samples for quality control. **(C)** Dot plot of DEGs in NK cell clusters from HCMV^{+}/HD (after exclusion of mitochondrial genes). **(D)** Violin plot with aggregated transcript counts of stem-like ISG signature (based on Wu *et al*., Cell. 2018) in CD56^{bright}, cCD56^{dim}, adCD56^{dim} I and adCD56^{dim} II NK cells from HCMV^{+}/HDs. Statistical analysis were performed via Kruskal-Wallis test, p<2.2^{e-16}, ****: p<0.0001. DEG, differentially expressed gene.

Figure S2

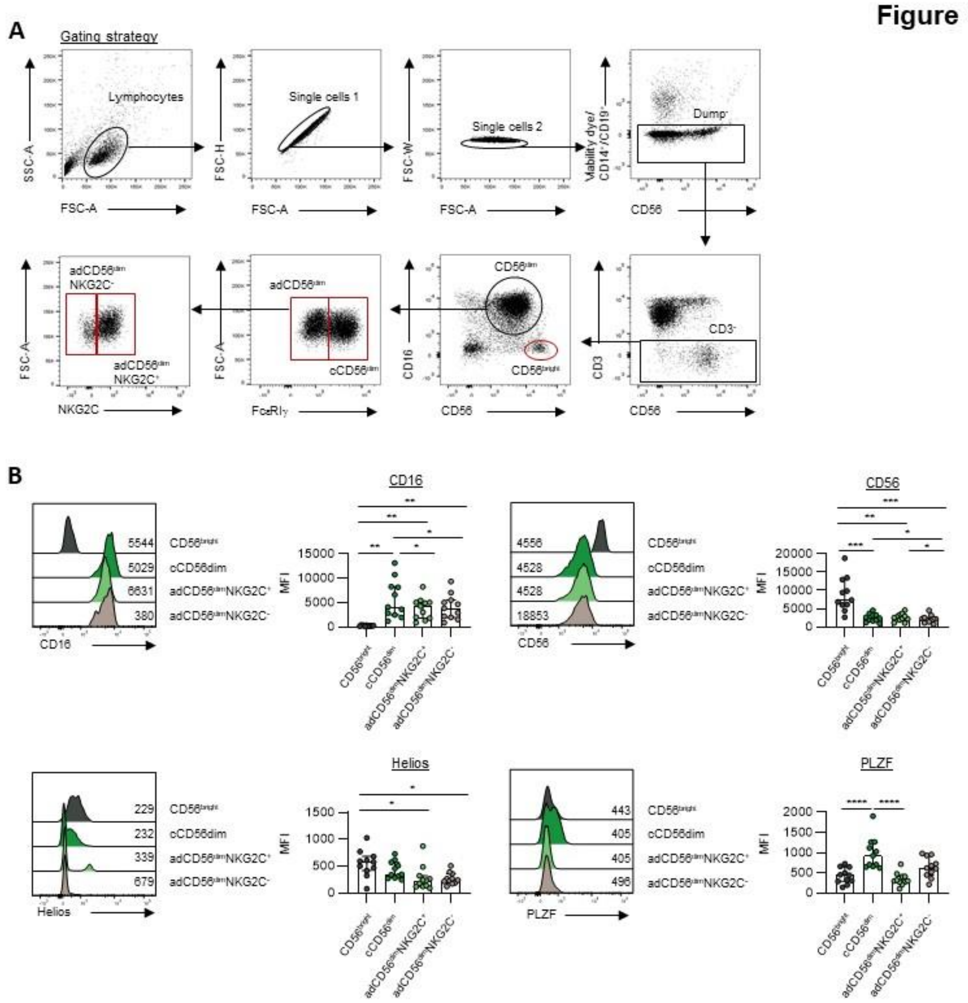

Figure S2: NK cell subset analysis on protein level, <u>related to STAR Methods.</u> (A) Gating strategy to define the NK cell clusters (CD56^{bright}, cCD56^{dim} (as CD56^{dim}FceRIg^{+}) and adCD56^{dim} (as CD56^{dim}FceRIg^{-}) via flow cytometry analysis. **(B)** Representative flow cytometry histograms and bar charts depicting co-expression analysis of CD16, CD56, FceRIg, Helios, PLZF and NKG2C of NK cells from HCMV^{+}/HD (n=11). Bar charts indicate the median value with IQR. Statistical analysis were performed via one-way repeated measures ANOVA (CD16 and CD56) and Friedman test (Helios and PLZF), *: p<0.05, **: p<0.01, ***: p<0.001, ****: p<0.0001.

Figure S3

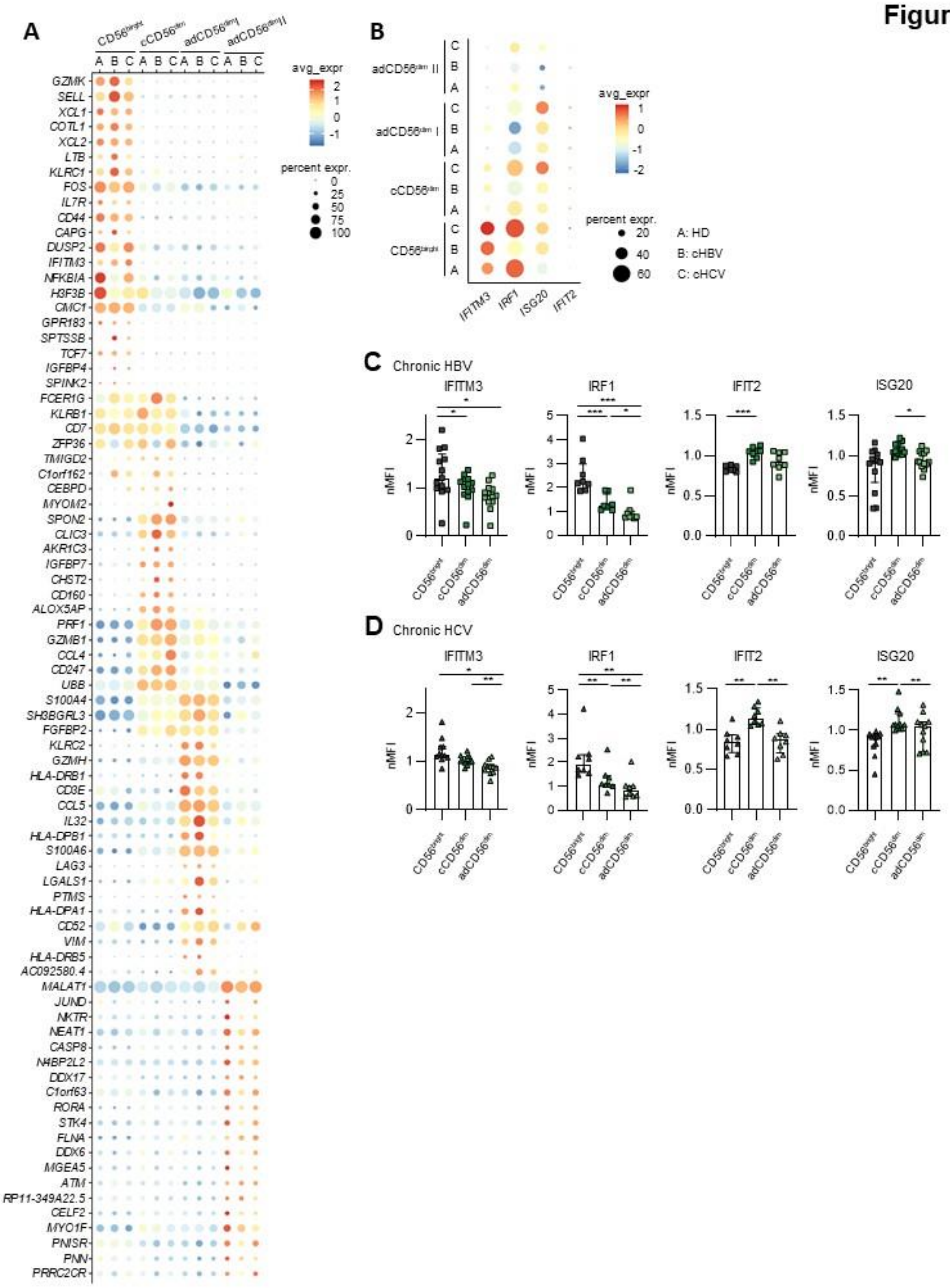

Figure S3: ISG expression of NK cells obtained from patients with chronic hepatitis virus infections, <u>related to Figure 2.</u> (A) Dot plot of DEGs in NK cell clusters from HCMV^{+}/HD, HCMV^{+}/cHBV- and HCMV^{+}/cHCV-infected patients (after exclusion of mitochondrial genes). **(B)** Dot plot with average expression of IFITM3, IRF1, IFIT2 and ISG20 (as surrogate markers) in NK cell clusters from HCMV^{+}/HD, HCMV^{+}/cHBV- and HCMV^{+}/cHCV-infected patients is shown. **(C-D)** Normalized MFI of IFITM3 IRF1 and IFIT2 and ISG20 on protein level in NK cell clusters obtained from **(C)** HCMV^{+}/cHBV- (n=8-13) and **(D)** HCMV^{+}/cHCV-infected patient (n=8-11). Bar charts indicate the median value with IQR. MFI of respective ISG expression was normalized to CD3^{-}CD56^{+} NK cells. Statistical significance was assessed by (C) ordinary one-way repeated measures ANOVA (IFITM3, IRF1, ISG20) and Friedmann test (IFIT2) and (D) one-way repeated measures ANOVA (IFITM3, IRF1, IFIT2) and Friedmann test (ISG20), $p>0.05$, *: $p<0.05$, **: $p<0.01$, ***: $p<0.001$. cHBV, chronic hepatitis B virus infection; cHCV, chronic hepatitis C virus infection.

Figure S4

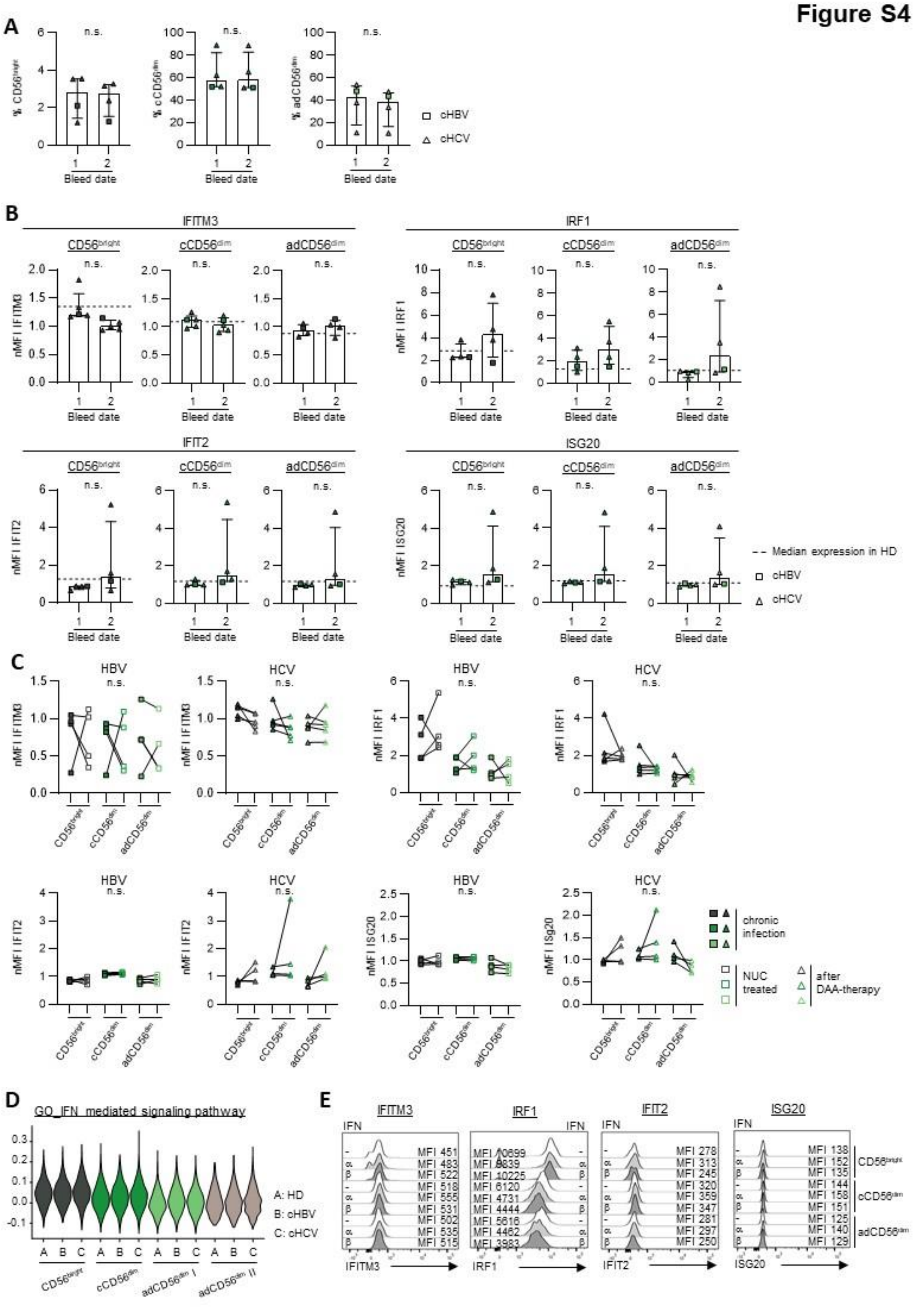

Figure S4: **Longitudinal analysis of ISG expression in NK cells during hepatitis virus infections, <u>related to Figure 3.</u> (A)** Bar graph with percentage of NK cell cluster during chronic phase of HBV or HCV infection. **(B-C)** Normalized MFI of IFITM3, IRF1, IFIT2 and ISG20 on protein level in NK cell clusters obtained from HCMV^{+}/cHBV- (n=1) or HCMV^{+}/cHCV-infected patients (n=3) **(B)** on two different time points during chronic infection and **(C)** in comparison to NUC treated (n=4) and after DAA-mediated clearance (n=4). **(D)** GO term enrichment analysis of IFN-mediated signaling pathway (GO:0140888) in the different NK cell clusters (dark gray: CD56^{bright}, green: cCD56^{dim}, light green: adCD56^{dim} I and beige: adCD56^{dim} II). **(E)** Representative flow cytometry histograms of ISG expression in NK cells with IFNα2 (light gray), IFNβ (gray) and without IFN stimulation (unstim.; white). Bar charts indicate the median value with IQR. Statistical significances were tested with (A) paired-t test, (B) Wilcoxon test (IFITM3/CD56bright, IFIT2/CD56bright, ISG20/cCD56dim) and paired t test and (C) Wilcoxon test (HBV: IFITM3/CD56^{bright}/CD56^{dim}, HCV: IRF1/CD56^{bright}/cCD56^{dim}, HCV: IFIT2/cCD56^{dim}/adCD56^{dim}) and paired t test , p>0.05. n.s., not significant. -: unstimulated. α: IFNα2. β: IFNβ.

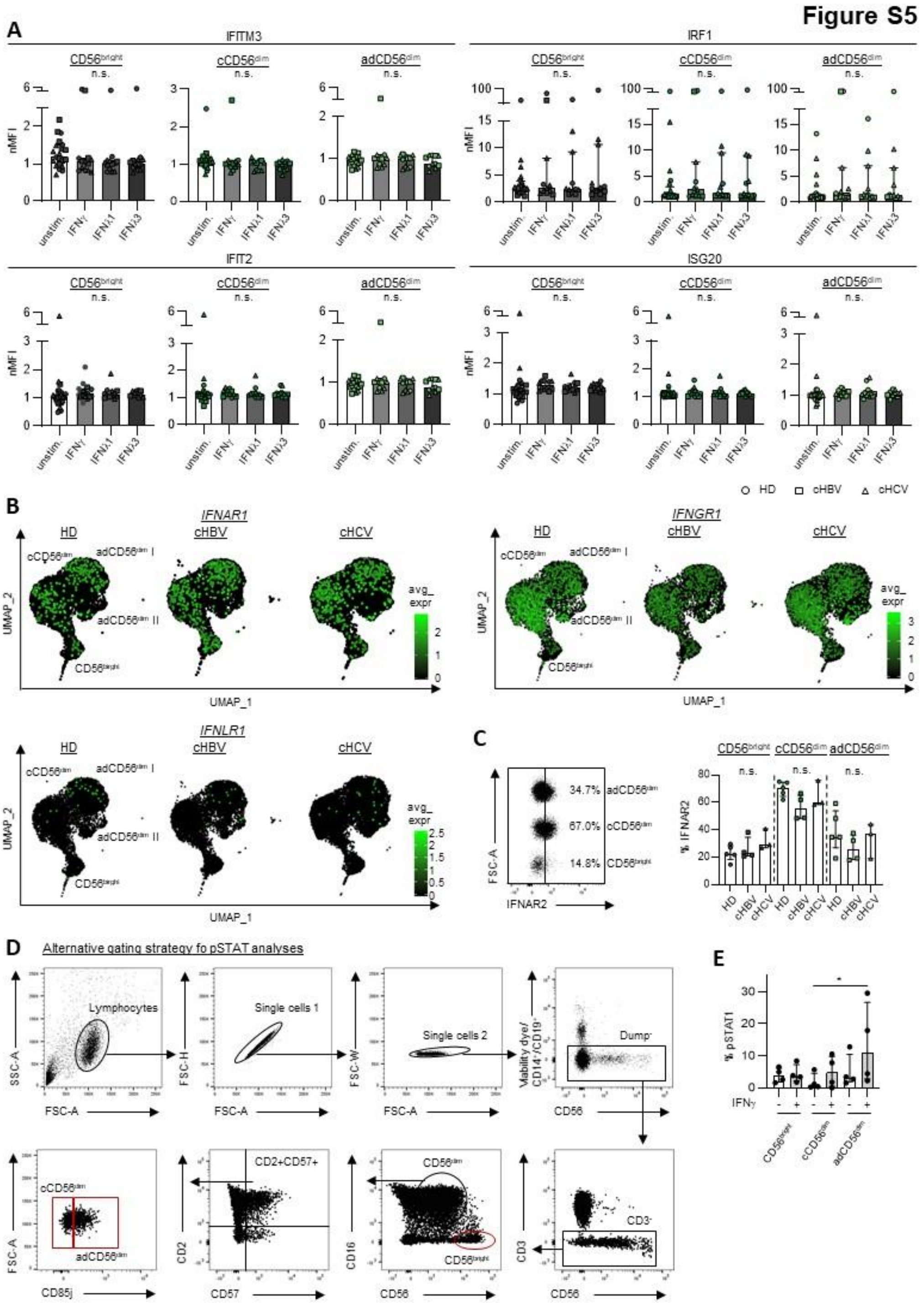

Figure S5: Little impact of type-II and –III IFNσ on ISG expression in NK cell, related to Figure 3. (A) Bar graphs depicting the normalized MFI of ISG expression upon IFNγ, IFNλ1 (gray), IFNλ3 (dark gray) or without (unstim.; white) IFN stimulation overnight in distinct NK cell clusters from HCMV$^+$/HD (n=3), HCMV$^+$/cHBV- (n=3) and HCMV$^+$/cHCV-infected patients (n=5). **(B)** Feature plots *of IFNAR1, IFNGR1* and *IFNLR1* expression in NK cell clusters from scRNAseq data. **(C)** Representative flow cytometry dot plots showing IFNAR2 expression in CD56^{bright}, cCD56^{dim} and adCD56^{dim} NK cells. Bar charts indicate frequencies of IFNAR2 expression in NK cell clusters from HCMV$^+$/HDs (n=5) and patients suffering from HCMV$^+$/cHBV (n=4) and HCMV$^+$/cHCV infection (n=3). **(D)** Gating strategy to define NK cell subsets (CD56^{bright}, cCD56^{dim} and adCD56^{dim}) on protein level via flow cytometry analysis for detection of pSTAT. The intracellular staining for FcεRIγ was not compatible with the staining protocol for phosphorylated STAT proteins. CD56^{dim} NK cell clusters were separated by the expression of the surface markers CD2, CD57 and CD85j. **(E)** Bar charts indicate frequencies of pSTAT1 expression with or w/o IFNγ stimulation in NK cell clusters from HCMV$^+$/cHCV-infected patients (n=4). Each dot represents one CD3$^-$CD56$^+$ NK cell. Bar charts indicate the median value with IQR. Statistical significances were tested with (A) Kruskal-Wallis test, (C) Kruskal-Wallis test (CD56^{bright}) and one-way repeated measures ANOVA (cCD56^{dim} and adCD56^{dim}) and (E) Friedman test, p>0.05. *: p<0.05, , n.s., not significant.

Figure S6

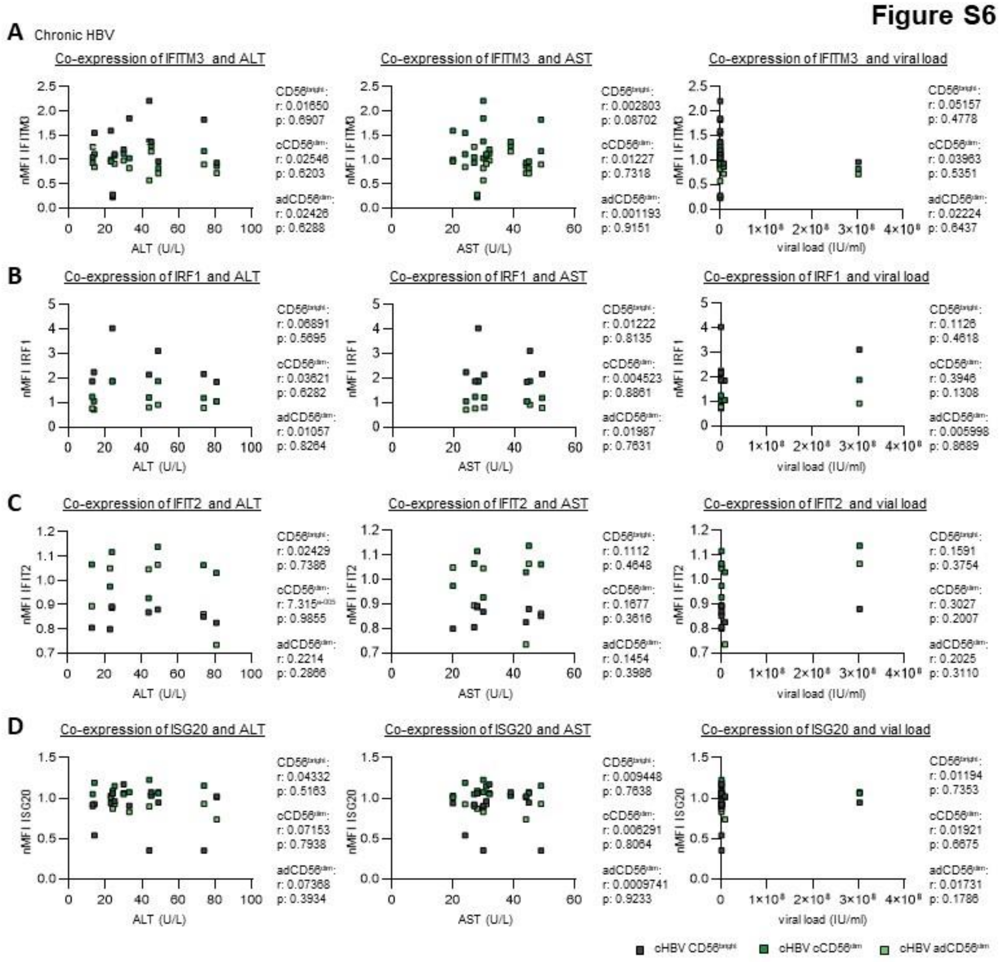

Figure S6: Correlation analysis of ISG expression in NK cells from patients with chronic HBV infection and clinical parameters, <u>related to Figure 3.</u> Correlation analysis of **(A)** IFITM3, **(B)** IRF1, **(C)** IFIT2 and **(D)** ISG20 in NK cell clusters from HCMV+/cHBV-infected patients (n=7-12) with AST, ALT and viral load. MFI of ISG expression was normalized to $CD3^{-}CD56^{+}$ NK cells. Statistical significance was assessed by simple linear regression. ALT, alanine aminotransaminase. AST, aspartate aminotransferase.

Figure S7

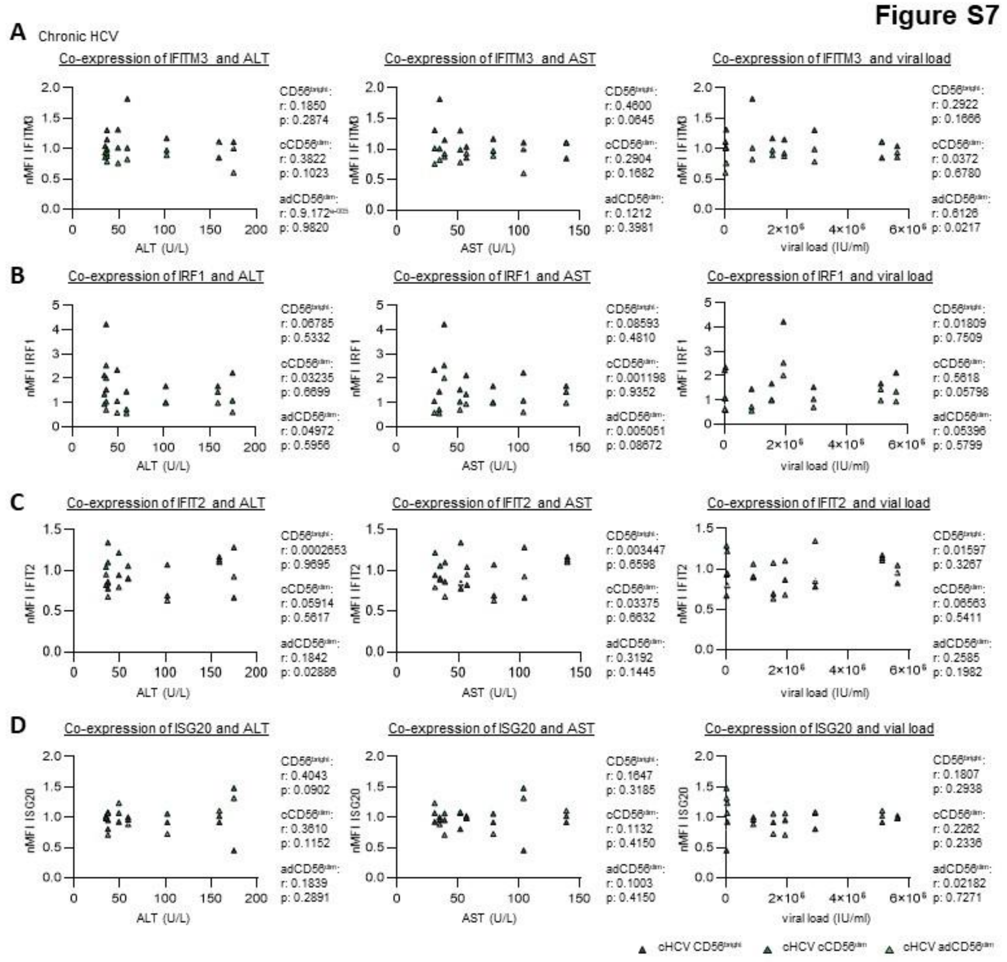

Figure S7: Correlation analysis of ISG expression in NK cells from patients with chronic HCV infection and clinical parameters, related to Figure 3. Correlation analysis of **(A)** IFITM3, **(B)** IRF1, **(C)** IFIT2 and **(D)** ISG20 in NK cell clusters from HCMV+/cHCV-infected patients (n=8) with AST, ALT and viral load. MFI of ISG expression was normalized to $CD3^{-}CD56^{+}$ NK cells. Statistical significance was assessed by simple linear regression. ALT, alanine aminotransaminase. AST, aspartate aminotransferase.

Figure S8

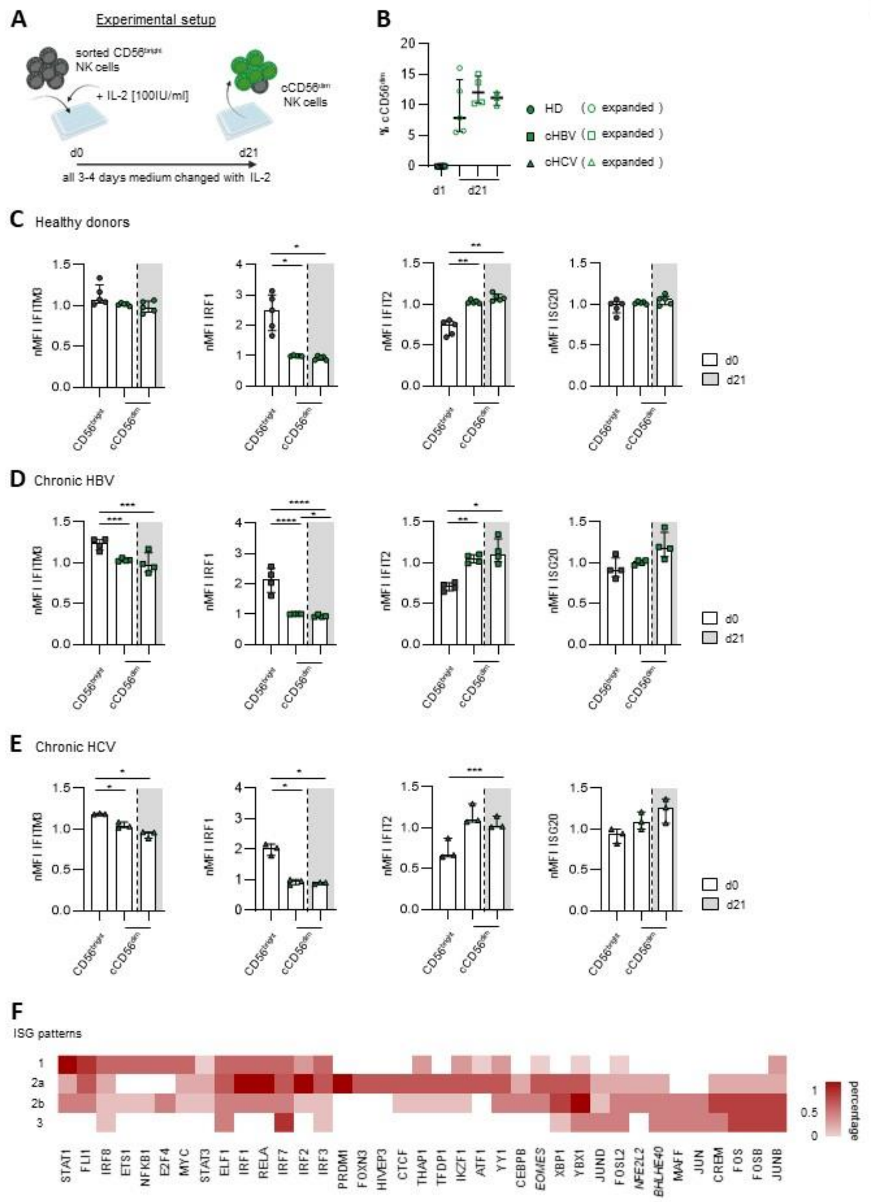

Figure S8: *In vitro* differentiation of NK cells and co-regulatory network analysis, <u>related to Figure 4.</u> **(A)** Schematic overview of the experimental setup of *in vitro* NK cell differentiation assays. Sorted CD56^{bright} NK cells were expanded with IL-2 for 21 days, afterwards comparative ISG expression analyses were performed in CD56^{bright} and cCD56^{dim} NK cells. **(B)** Bar graph indicated the percentage of cCD56^{dim} NK cells from HCMV^{+}/HD (n=5), HCMV^{+}/cHBV- (n=4) and HCMV^{+}/cHCV-infected patients at day 0 and day 21 in *in vitro* differentiation assay. **(C-E)** Statistical graphs depicting ISG expression in CD56^{bright} and cCD56^{dim} NK cells of **(C)** HCMV^{+}/HD (n=5), **(D)** HCMV^{+}/cHBV- (n=4) and **(E)** HCMV^{+}/cHCV-infected patients (n=3) *ex vivo* (white) and *in vitro* after differentiation at day 21 (light grey) measured by flow cytometry. **(F)** Binary heatmap showing the mean percentage of co-regulation of the previously defined four ISG sets with distinct transcription factors based on NK cell scRNAseq data merged from HCMV^{+}/HDs, HCMV^{+}/cHBV- and HCMV^{+}/cHCV-infected patients. Bar charts indicate the median value with IQR. Statistical significances were tested with ordinary one-way repeated measures ANOVA, p>0.05, *: p<0.05, **: p<0.01, ***: p<0.001, ****: p<0.0001.

Supplemental Tables

Table S1: Study cohort of HCMV$^+$ health donors, related to STAR Methods

Patient ID	HCMV	Sex	Age
HD1	pos	f	57
HD2	pos	f	26
HD3	pos	m	BL1:31<br>BL2:32
HD4	pos	f	27
HD5	pos	f	37
HD6	pos	f	BL1:28<br>BL2:29
HD7	pos	f	30
HD8	pos	f	30
HD9	pos	m	35
HD10	pos	m	45
HD11	pos	m	34
HD12	pos	m	33
HD13	pos	m	55
HD14	pos	f	47
HD15	pos	f	BL1:31<br>BL2:32
HD16	pos	m	23
HD17	pos	m	21
HD18	pos	f	30

Abbreviations: HCMV: human cytomegalovirus, F: female, M: male, BD: bleed date

Table S2: Study cohort of HCMV^{+}/cHBV-infected patients, related to STAR Methods

Patient ID	HCMV	Sex	Age	Gt	Clinic. phase	Therapy	VL (IU/ml)	AST (U/L)	ALT (U/L)	HBe Ag	HBs Ag
HBV1	pos	m	35	D	chronic	naive	>1000	33	55	pos	pos
HBV2	pos	m	34	E	chronic	naive	n.d.	143	223	pos	pos
HBV3	pos	f	35	A	chronic	naive	2298	n.d.	32	n.d.	pos
HBV4	pos	f	33	n.d.	chronic	naive	327	n.d.	22	n.d.	pos
HBV5	pos	f	34	n.d.	chronic	naive	2625	24	14	neg	pos
HBV6	pos	m	37	n.d.	chronic	naive	427	30	44	n.d.	pos
HBV7	pos	m	35	n.d.	chronic	naive	418484	49	74	pos	pos
HBV8	pos	f	36	D	chronic	naive	3214	20	23	neg	pos
HBV9	pos	f	BL1:43<br>BL2:45	D	chronic	naive	BL1:3214<br>BL2:1306	BL1:30<br>BL2:29	BL1:43<br>BL2:34	neg	pos
HBV10	pos	m	59	n.d.	chronic	naive	59	32	33	neg	pos
HBV11	pos	m	39	n.d.	chronic	naive	277	31	25	n.d.	pos
HBV12	pos	f	18	C	chronic	naive	387669	39	45	pos	pos
HBV13	pos	f	35	n.d.	chronic	naive	6003	22	22	n.d.	pos
HBV14	pos	f	45	n.d.	chronic	naive	19	59	90	neg	pos
HBV15	pos	f	26	D	chronic	naive	302635258	45	49	pos	pos
HBV16	pos	m	BL1:31<br>BL2:33	D	chronic	BL1:naive<br>BL2:NUC treated	BL1:7800265<br>BL2:13	BL1:44<br>BL2:48	BL1:81<br>BL2:126	pos	pos
HBV17	pos	f	BL1:35<br>BL2:37	n.d.	chronic	BL1:naive<br>BL2:NUC treated	BL1:25347<br>BL2:neg	BL1:27<br>BL2:21	BL1:13<br>BL2:41	n.d.	pos
HBV18	pos	m	BL1:57<br>BL2:59	n.d.	chronic	BL1:naive<br>BL2:NUC treated	BL1:23335<br>BL2:neg	BL1:28<br>BL2:24	BL1:41<br>BL2:31	n.d.	pos
HBV19	pos	m	BL1:38<br>BL2:41	n.d.	chronic	BL1:chronic<br>BL2:NUC treated	BL1:6540<br>BL2:neg	n.d.	n.d.	neg	pos

Abbreviations: HCMV: human cytomegalovirus, F: female, M: male, cHBV: chronic hepatitis B virus, Gt: genotype; Clinic. Phase: clinical phase; VL: viral load, ALT: alanine aminotransaminase, AST: aspartate aminotransferase, pos: positive, neg: negative, n.d.: not done, BD: bleed date, NUC: nucleos(t)ide analogue

Table S3: Study cohort of HCMV+/cHCV-infected patients, related to STAR Methods

Patient ID	HCMV	Sex	Age	Gt	clinical phase	Therapy	VL (IU/ml)	AST (U/L)	ALT (U/L)
HCV1	pos	m	27	1b	chronic	naive	5059859	50	75
HCV2	pos	m	44	1a	chronic	naive	1351628	47	69
HCV3	pos	m	29	1b	chronic	naive	743436	50	69
HCV4	pos	f	30	1b	chronic	naive	28979	31	49
HCV5	pos	m	BL1:31<br>BL2:33	3a	chronic	naive	BL1:5121998<br>BL2:210355	BL1:139<br>BL2:n.d.	BL1:159<br>BL2:194
HCV6	pos	f	BL1:63<br>BL2:70<br>BL3:70	1b	BL1:chronic<br>BL2:chronic<br>BL3:cured	BL1:naive<br>BL2:naive<br>BL3:after DAA therapy	BL1:n.d.<br>BL2:2920917<br>BL3: neg	BL1:58<br>BL2:52<br>BL3:23	BL1:51<br>BL2:37<br>BL3:16
HCV7	pos	m	BL1:37<br>BL2:38<br>BL3:38	1a	BL1:chronic<br>BL2:chronic<br>BL3:cured	BL1:naive<br>BL2:naive<br>BL3:after DAA therapy	BL1:n.d.<br>BL2:n.d.<br>BL3: neg	BL1:53<br>BL2:62<br>BL3:20	BL1:155<br>BL2:167<br>BL3:26
HCV8	pos	m	31	1b	chronic	naive	104	104	175
HCV9	pos	m	BL1:35<br>BL2:37	3a	chronic	naive	BL1:884011<br>BL2:n.d.	BL1:35<br>BL2:33	BL1:59<br>BL2:42
HCV10	pos	f	48	1a	chronic	naive	n.d.	74	155
HCV11	pos	m	50	3a	chronic	naive	1588000	50	76
HCV12	pos	f	32	1b	chronic	naive	n.d.	n.d.	n.d.
HCV13	pos	f	67	1b	chronic	naive	n.d.	32	43
HCV14	pos	f	58	1b	chronic	naive	5622273	57	35
HCV15	pos	f	75	4a	chronic	naive	136194	29	22
HCV16	pos	m	62	n.d.	chronic	naive	n.d.	n.d.	n.d.
HCV17	pos	m	56	n.d.	chronic	naive	3739	54	36
HCV18	pos	m	64	1b	chronic	naive	647	29	23
HCV19	pos	f	BL1:71<br>BL2:72	1b	BL1:chronic<br>BL2:cured	BL1:naive<br>BL2:after DAA therapy	BL1:1543939<br>BL2:neg	BL1:79<br>BL2:26	BL1:102<br>BL2:16
HCV20	pos	m	BL1:46<br>BL2:47	3a	BL1:chronic<br>BL2:cured	BL1:naive<br>BL2:after DAA therapy	BL1:1930622<br>BL2:neg	BL1:39<br>BL2:29	BL1:37<br>BL2:20

Abbreviations: HCMV: human cytomegalovirus, F: female, M: male, cHCV: chronic hepatitis C virus, Gt: genotype; VL: viral load, ALT: alanine aminotransaminase, AST: aspartate aminotransferase, pos: positive, neg: negative, n.d.: not done, BD: bleed date, DAA: direct-acting antiviral
